# Extranodal lymphoma: pathogenesis, diagnosis and treatment

**DOI:** 10.1186/s43556-023-00141-3

**Published:** 2023-09-18

**Authors:** Hua Yang, Yang Xun, Chao Ke, Kensuke Tateishi, Hua You

**Affiliations:** 1 Department of Basic Medicine and Biomedical Engineering, School of Medicine, Foshan University, Foshan, 528000 China; 2 Department of Neurosurgery and Neuro-Oncology, Sun Yat-Sen University Cancer Center, State Key Laboratory of Oncology in South China, Collaborative Innovation Center for Cancer Medicine, Guangzhou, 510060 China; 3 Department of Neurosurgery, Graduate School of Medicine, Yokohama City University, Yokohama, 2360004 Japan; 4 Laboratory for Excellence in Systems Biomedicine of Pediatric Oncology, Department of Pediatric Hematology and Oncology, Chongqing Key Laboratory of Pediatrics, Ministry of Education Key Laboratory of Child Development and Disorders, China International Science and Technology Cooperation base of Child development and Critical Disorders, National Clinical Research Center for Child Health and Disorders, Children’s Hospital of Chongqing Medical University, Chongqing, 401122 China

**Keywords:** Extranodal lymphoma, Signaling pathway, Diagnosis, Treatment, Prognosis

## Abstract

Approximately 30% of lymphomas occur outside the lymph nodes, spleen, or bone marrow, and the incidence of extranodal lymphoma has been rising in the past decade. While traditional chemotherapy and radiation therapy can improve survival outcomes for certain patients, the prognosis for extranodal lymphoma patients remains unsatisfactory. Extranodal lymphomas in different anatomical sites often have distinct cellular origins, pathogenic mechanisms, and clinical manifestations, significantly influencing their diagnosis and treatment. Therefore, it is necessary to provide a comprehensive summary of the pathogenesis, diagnosis, and treatment progress of extranodal lymphoma overall and specifically for different anatomical sites. This review summarizes the current progress in the common key signaling pathways in the development of extranodal lymphomas and intervention therapy. Furthermore, it provides insights into the pathogenesis, diagnosis, and treatment strategies of common extranodal lymphomas, including gastric mucosa-associated lymphoid tissue (MALT) lymphoma, mycosis fungoides (MF), natural killer/T-cell lymphoma (nasal type, NKTCL-NT), and primary central nervous system lymphoma (PCNSL). Additionally, as PCNSL is one of the extranodal lymphomas with the worst prognosis, this review specifically summarizes prognostic indicators and discusses the challenges and opportunities related to its clinical applications. The aim of this review is to assist clinical physicians and researchers in understanding the current status of extranodal lymphomas, enabling them to make informed clinical decisions that contribute to improving patient prognosis.

## Introduction

Approximately 30% of lymphomas arise from sites other than the lymph nodes, spleen or bone marrow [1]. The prevalence of extranodal lymphoma has increased over the past decade [2]. There are two main types of lymphoma exist: B-cell lymphoma and T-cell lymphoma, with B-cell lymphomas being more prevalent than T-cell lymphomas [3]. Common sites of extranodal lymphoma include the gastrointestinal tract, head and neck, skin/soft tissue, central nervous system (CNS) [4, 5]. Different sites of extranodal lymphoma often have unique cellular origins, genetic abnormalities, and clinical behaviour [6].

The diagnosis of extranodal lymphoma necessitates a comprehensive assessment encompassing clinical symptoms, physical examination findings, and laboratory tests. Commonly utilized diagnostic tools comprise imaging studies, such as X-rays, CT scans, MRIs, and PET scans, enabling the identification of the site and extent of lymphoma involvement. Biopsy procedures, including needle biopsies or surgical interventions, are employed to obtain tissue samples, which serve as definitive evidence for lymphoma diagnosis. Immunohistochemistry and genetic tests may be conducted to determine the specific subtype and prognosis of the lymphoma. Furthermore, given the potential involvement of the bone marrow in extranodal lymphoma, a bone marrow biopsy is often performed to evaluate disease spread and facilitate the selection of appropriate treatment strategies. This comprehensive diagnostic approach provides valuable insights into the nature and extent of extranodal lymphoma, aiding in the formulation of effective management plans [7, 8].

The treatment of extranodal lymphoma depends on aspects such as subtype, stage of the disease and the patient's overall health. Conventional treatments include chemotherapy, radiation therapy, targeted therapy, and immunotherapy. Chemotherapy stands as the cornerstone of treatment for the majority of extranodal lymphomas [9] and radiation therapy represents a localized therapeutic modality [10]. In addition, autologous hematopoietic stem cell transplantation (ASCT) may also be an effective salvage measure for extra-nodal lymphoma.

As the diagnosis and treatment of extranodal lymphoma are influenced by its different pathogenesis at different anatomical sites, it is necessary to discuss the pathogenesis of extranodal lymphoma at different sites separately. This review will summarize the common key signaling pathways and intervention treatments in extranodal lymphomas. Furthermore, we thoroughly explore the pathogenesis, diagnosis, and treatment strategies of MALT, NKTCL, mycosis fungoides (MF), and PCNSL, which are the most notable types of extranodal lymphomas occurring in the gastrointestinal tract, head and neck region, skin, and CNS. Besides, considering that PCNSL is one of the lymphomas with the poorest prognosis and there are few articles synthesizing its prognostic indicators, we have comprehensively summarized the prognostic markers of PCNSL and discussed the challenges and opportunities related to clinical applications. This review will contribute to enhancing our understanding of extranodal lymphomas and provide valuable insights for future clinical decision-making.

## Signaling pathways and interventional therapy in extranodal lymphoma

**Fig. 1:**
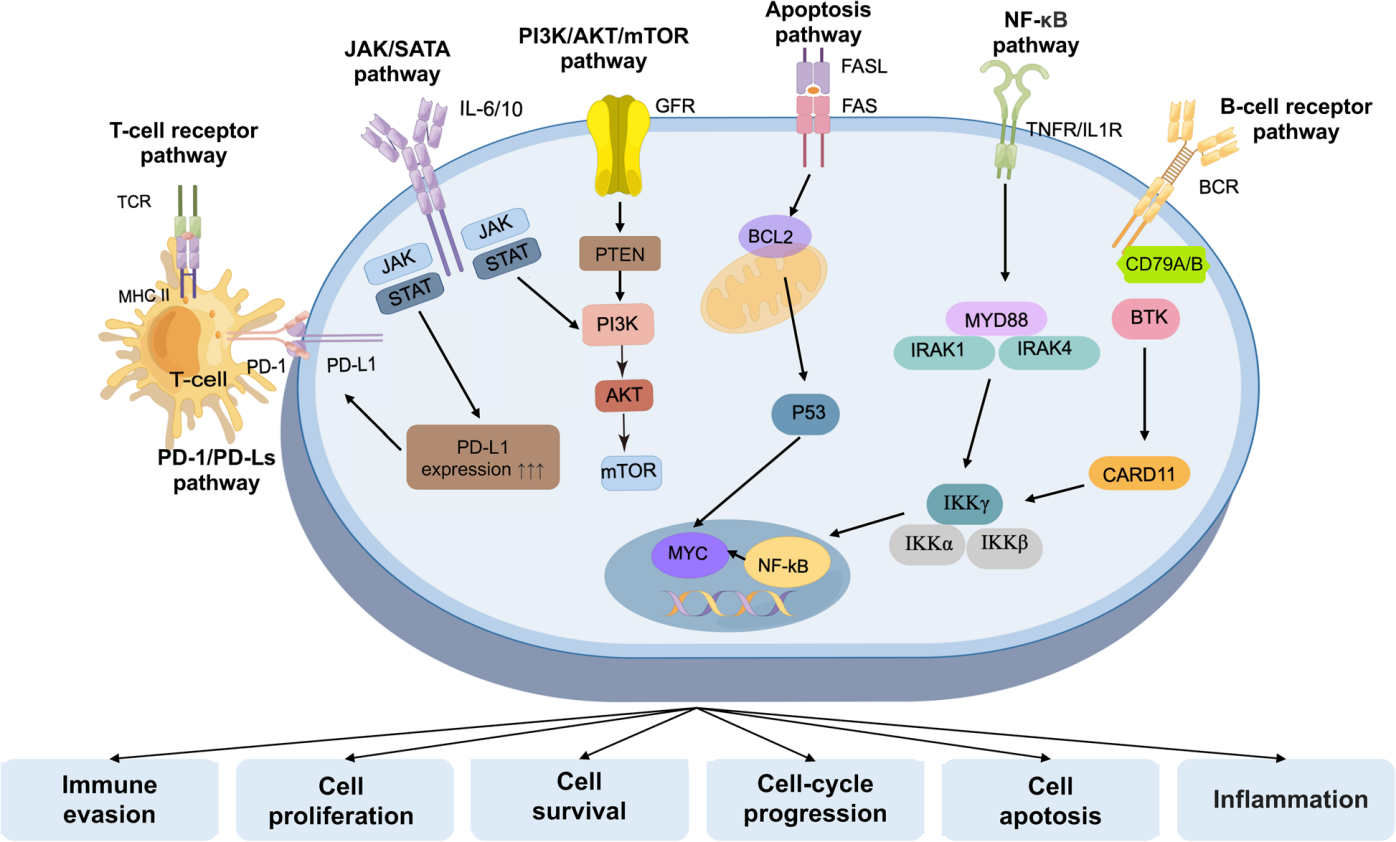
Common key signaling pathways in extranodal lymphoma

A variety of signaling pathways have been implicated in the pathogenesis of extranodal lymphomas. While different sites of extranodal lymphoma may exhibit distinct signaling pathways, there are still common key signaling pathways shared among them (Fig. 1). These include the NF-κB pathways, Janus-associated kinase/signal transducer and activator of transcription (JAK/STAT) pathways, phosphatidylinositol 3-kinase (PI3K)/ protein kinase B (Akt)/ mammalian target of rapamycin (mTOR) pathway, apoptosis pathway, programmed death-1/programmed death-ligands (PD-1/PD-Ls) pathway, and Cell receptor signaling pathway. Table 1 summarizes the common signal pathway inhibitors currently under clinical investigation for extranodal lymphoma.

**Table 1:** Overview of signaling pathway inhibitors undergoing clinical studies in extranodal lymphoma

Cancer Type	Signaling Pathway	Drugs
PCNSL	PD-1/PD-Ls	Camrelizumab, Durvalumab, F520, GNC-038, Nivolumab, Pembrolizumab, Penpulimab, Sintilimab
PCNSL	B-cell receptors	Acalabrutinib, Ibrutinib, NX-2127, NX-5948, Orelabrutinib, Tirabrutinib, Zanubrutinib
PCNSL	PI3K/AKT/mTOR	Bimiralisib, Buparlisib, Emavusertib, Paxalisib
MALT	PD-1/PD-Ls	Pembrolizumab
MALT	B-cell receptors	AC-676, Acalabrutinib, AS-1763, BGB-16673, HMPL-760, Ibrutinib, NX-2127, Orelabrutinib, Zanubrutinib
MALT	PI3K/AKT/mTOR	BGB-10188, BGB-16673, BR101801, Copanlisib, Duvelisib, GS-9901, HMPL-689, HMPL-760, IBI376, Idelalisib, NX-2127, Orelabrutinib, SHC014748M, Umbralisib, YY-20394, Zandelisib, Zanubrutinib
MALT	NF-κB	BGB-21447, CC-99282, LP-168, VAY736, XL114
MALT	JAK/STAT	CpG-STAT3 siRNA CAS3/SS3
MALT	Apoptosis	L-Bcl-2 antisense oligonucleotide
PTCL	PD-1/PD-Ls	AB-101, F-520, GB-226, ONO-4685, Sintilimab, Tislelizumab
PTCL	NF-κB	Copanlisib, Duvelisib, HMPL-689, IOA-244, Linperlisib, Parsaclisib, SHC014748M, TQ-B3525, YY-20394
PTCL	JAK/STAT	AZD4205, KT-333
PTCL	Apoptosis	ASTX660, L-Bcl-2 antisense oligonucleotide, Tolinapant
NK-T	PD-1/PD-Ls	IMC-001, SHR-1210, Sintilimab, Sugemalimab, Tislelizumab, Toripalimab
NK-T	NF-κB	YY-20394
NK-T	JAK/STAT	Ruxolitinib, Tofacitinib

*MALT* Mucosa-associated lymphoid tissue lymphomas, *NKTCL-NT* Natural killer/T-cell lymphoma, nasal type, *PCNSL* Primary central nervous system lymphoma, *PTCL* Peripheral T cell lymphoma

### NF-κB pathway

The NF-κB pathway is constitutively activated and plays a role in cell survival, proliferation, and immune responses in various types of extranodal lymphoma [11–15]. Both the classical NF-κB pathway, activated by the tumor necrosis factor-α receptor (TNFR) 1, interleukin1 receptor (IL1R), toll-like receptor (TLR), T-cell receptors (TCR), B-cell receptors (BCR), and growth factor receptors (GFR), and the alternative NF-κB pathway, activated by TNFR, CD40 and B-cell activating factor (BAFF), play roles in cell survival, proliferation, inflammation, and immune. In lymphoma, both pathways are constitutively activated and contribute to oncogenic events [16]. Abnormalities in the NF-κB pathway and its upstream or downstream pathways, such as the BCR (B-cell receptor) or TLR pathways, are important mechanisms in the development of lymphomas. Mutations or chromosomal translocations in *CARD11*, *CD79A/B* and *myeloid differentiation primary response 88 (MYD88)* contribute to the activation of the NF-κB pathway [15, 17]. Currently, inhibitors targeting upstream targets of the NF-κB pathway, such as Bruton's Tyrosine Kinase (BTK) inhibitors [18], receptor proximal kinases in NF-κB (like interleukin-1 receptor associated kinase 4 inhibitors) [17] and CD30 inhibitors (typical examples include brentuximab vedotin) [17], have been found to be effective in treating extranodal lymphomas.

### JAK/STAT pathway

The JAK/STAT pathway mediates the transmission of signals from cytokines and growth factors. Dysregulation of this pathway has been observed in extranodal lymphomas, including mucosa-associated lymphoid tissue (MALT) lymphomas, natural killer/T-cell lymphoma (nasal type, NKTCL-NT) [13, 19], primary central nervous system lymphoma (PCNSL) [20] and peripheral T cell lymphoma (PTCL) [21, 22]. The JAK/STAT pathway is associated with immune homeostasis, inflammation, cell proliferation, apoptosis and differentiation in extranodal lymphoma [21]. Abnormalities in JAK and STAT have been identified as distinct characteristics of lymphoma. Besides, JAK/STAT pathway may play a role in upregulating PD-L1 and PD-L2 expression in extranodal lymphomas [23]. Inhibitors targeting JAK kinases have shown promising results. The JAK2/FLT3 fusion inhibitor pacritinib has demonstrated preclinical safety and efficacy [22]. The JAK3 inhibitor tofacitinib has shown therapeutic effects in PTCL [24] and NKTCL [19]. A small molecule inhibitor of STAT3 called Stattic can also be effective by inducing apoptosis or inhibiting cell proliferation in NKTCL cells [19]. Targeting the JAK/STAT pathway may provide new treatment options for patients with extranodal lymphomas.

### PI3K/AKT/mTOR pathway

The PI3K/AKT/mTOR pathway plays an oncogenic role in extranodal lymphomas. Isoforms p110δ and p110γ belong to the Class I kinase of the PI3K family play a crucial role in the development, proliferation, migration, cytokine secretion, and other cellular functions of B-cells [25, 26], T-cells [26, 27], and NK-cells [28, 29]. Novel PI3K inhibitors (idelalisib, buparlisib, duvelisib and copanlisib) have recently demonstrated promise for the treatment of MALT [30–32], PCNSL [33], NKTCL [34] and PTCL [27, 34, 35].

AKT and PTEN are key proteins in the PI3K pathway, acting as positive and negative regulators, respectively. When PTEN is inactivated, it leads to an increase in AKT and mTOR activity, which in turn promotes tumor growth and other pathological changes [17]. Therefore, it is reasonable to target the PI3K/AKT/mTOR pathway for the treatment of PTEN-deficient extranodal lymphomas. Several agents such as AKT inhibitors (MK-2206) [36, 37] and pan-PI3K inhibitor (buparlisib) [38] are under clinical evaluation.

The mTOR is also a key protein kinase in the PI3K/AKT/mTOR pathway, and it serves as a structural unit for mTORC1 and mTORC2 complexes. Upon receiving phosphorylation signals from upstream AKT, activated mTORC1 and mTORC2 carry out their respective functions, positively regulating cell survival, induction of cell cycle arrest, and negatively regulating autophagy [39]. The current focus of research is primarily on first-generation mTOR inhibitors. Among them, temsirolimus and everolimus are typical mTORC1 inhibitors. They can be used alone or in combination with rituximab and have been shown to have favorable efficacy in PCNSL [40, 41], PTCL [42, 43], MZL [40] and NKTCL [44].

### Apoptosis pathway

The apoptosis pathway dysregulation in extranodal lymphomas is often due to abnormalities in key regulators like BCL2, p53, and MYC [45–47]. For example, upregulation of P53 may inhibit apoptosis in NKTCL [45, 46]. Overexpression of MYC and BCL2 is frequently seen in patients with B-cell extranodal lymphomas [47, 48] and PTCL [49, 50]. BCL2 inhibitors like venetoclax [49] and obatoclax [50] can restore apoptosis. MYC inhibitors and p53 reactivate drugs are being explored. Currently, inhibitors targeting BCL2, p53 and MYC aim to restore apoptosis in lymphoma cells by reactivating programmed cell death mechanisms.

### PD-1/PD-Ls pathway

Extranodal lymphoma cells can exploit the PD-1/PD-Ls pathway to avoid immune surveillance by modulating T-lymphocyte activity [51]. PD-1, PD-L1 and PD-L2 were found to be overexpressed in B-cell and T-cell extranodal lymphomas and their tumor microenvironment [52]. The application of immune checkpoint inhibitors in lymphoma is receiving increasing attention. Checkpoint inhibitor antibodies blocking PD-1 (nivolumab, pembrolizumab and Sintilimab) or PD-L1 (atezolizumab, avelumab) have been classified as Level 3 evidence for use in salvage therapy for extranodal lymphoma, including MALT [52], NKTCL [51–55], PCNSL [56], PTCL [52, 54].

### Cell receptor pathway

The B-cell receptor (BCR) pathway is a crucial mechanism involved in the immune response. It is characterized by the activation of CD79A/CD79B heterodimers, which transmit antigen-stimulated signals from the cell membrane to the cytoplasm. The persistent activation of BCR pathway relies on the phosphorylation of immunoreceptor tyrosine-based activation motifs by Src family kinases, leading to the recruitment and activation of spleen tyrosine kinase. This activation triggers downstream signaling pathways, including PI3K/AKT/mTOR, NF-κB, and MAPK. CD79A/B and BTK play critical roles in this process, and their dysregulation has been implicated in B-cell extranodal lymphoma such as PCNSL [57, 58], MALT [59, 60]. Inhibition of BTK, with drugs like ibrutinib, has shown promising efficacy against these malignancies by disrupting BCR pathways and downstream NF-κB pathways.

The T-cell receptor (TCR) plays a key role in the pathogenesis of PTCL by providing "signal 1" through engagement with antigen peptides presented on major histocompatibility complex (MHC) molecules for lymphoma cell growth and survival [61]. Targeting TCR signaling, like Src family kinase inhibitors dasatinib is effective in treating PTCL. Dasatinib demonstrated an overall response rate of 29% in relapsed/refractory PTCL [62]. However, no TCR pathway inhibitor drug has been approved by the FDA. This may be due to the fact that the efficacy and safety of TCR pathway inhibitors require further study [63].

## Gastric mucosa-associated lymphoid tissue Lymphoma

### Pathogenesis

Gastric lymphoma is the most common form of extranodal lymphoma, accounting for 30–40% of all extra-nodal lymphomas. Histopathologically, MALT lymphoma is the most common primary gastric lymphoma subtype [64].

The majority of patients (80–90%) with gastric MALT lymphoma are infected with H. pylori (HP) [65]. The development of gastric MALT lymphoma is closely associated with HP-mediated regulation of T cells, HP-induced cytokines and chemokines, HP antigen stimulation, and s mediation of signaling molecules. T-cell responses induced by HP infection play a critical role in tumor growth and progression. In the early stages of gastric MALT lymphoma development, HP-stimulated infiltrating T cells promote the proliferation and differentiation of B lymphoma cells. This process involves CD40 signaling, secretion of Th2-type cytokines (such as interleukin-4, interleukin-5 and interleukin-10) upon exposure to HP antigens [66]. Moreover, alterations such as the loss of CXCR4 [67] and upregulation of CXCR7, BCA-1 and its receptor CXCR5 [68] are also involved in the development of gastric MALT lymphoma. Activation of phospho-Src homology-2 domain-containing phosphatase and HP CagA-mediated signaling molecules further promote B-cell proliferation [69]. Chronic infection often contributes to gastric MALT lymphoma by inducing aberrant B cell survival and proliferation through BCR pathway [70]. PI3K pathway is critical for the proliferation and survival of malignant B cells [71]. Interestingly, HP-negative MALT lymphomas have shown a high frequency of positive t(11;18) (q21;q21) [72]. This translocation event leads to the formation of a fusion protein called API2-MALT1, which in turn activates the transcription factor NF-kappa through enhanced IKK gamma polyubiquitination [73]. The above findings suggest that t(11;18)(q21;q21) may be a major contributor to the development of gastric MALT lymphoma and is associated with a poor prognosis [73].

### Diagnosis

In addition to routine physical examinations, blood tests, biochemistry, enhanced whole-body CT scans, and endoscopy can also be used as part of the pre-treatment evaluation of gastric MALT lymphoma. Endoscopy is an indispensable tool for the initial diagnosis and follow-up of gastric MALT lymphoma cases and for obtaining biopsy specimens [74]. The urea breath test can rapidly detect the presence of HP infection and can also assist in the repeated evaluation of the effectiveness of anti-HP treatment [75]. HBV [76] and HCV [77] testing not only aids in the diagnosis of certain types of gastric MALT lymphoma but may also serve as a therapeutic target. Gastric MALT lymphoma diagnosis depends on pathological diagnosis and all pathological specimens should be routinely tested by immunohistochemistry (IHC). The typical immunophenotypes of gastric MALT lymphoma are CD5-, CD10-, CD20 + , CD21-/ + , CD23-/ + , CD43-/ + , cyclin D1- and MNDA ± [8]. Detection of the translocation should also aid in the clinical management of patients with gastric MALT lymphoma. HP-negative gastric MALT lymphoma can be detected by reverse transcription-PCR or FISH and (t 11;18) is often indicative of advanced disease and poor anti-HP efficacy [78].

### Treatments

Anti-HP therapy is highly recommended for patients diagnosed with limited gastric MALT lymphoma and confirmed positive for HP infection [79]. Anti-HP therapy results in remission in 60–80% of patients, even in HP-negative patients [65]. For patients who are t(11;18)(q21;q21) positive, have residual tumors after anti-Hp therapy, experience symptoms such as concurrent bleeding, or are not suitable candidates for HP treatment, radiotherapy is frequently employed as a salvage treatment [78, 80]. Rituximab in combination with chemotherapy is the usual treatment modality for stage III/IV gastric MALT lymphoma that has failed local radiotherapy without B symptoms, bleeding, blood cell depletion, large masses or rapid tumor progression [81]. If the above treatments fail, new targeted drugs may be considered. The BTK inhibitor, ibrutinib, provides a chemotherapy-free treatment alternative for patients diagnosed with gastric MALT lymphoma. Remarkably, single-agent ibrutinib therapy has shown durable responses and a favorable benefit-risk profile in patients with gastric MALT lymphoma who have received prior treatment [70]. The PI3K inhibitor copanlisib has demonstrated significant efficacy and a manageable safety profile in patients with relapsed/refractory gastric MALT lymphoma who have received intensive treatment, and may be a salvage treatment option for patients [71].

## Mycosis fungoides

### Pathogenesis

Cutaneous lymphoma most commonly originates from T-cells [82]. Cutaneous T-Cell Lymphoma (CTCL) is broadly classified as a type of PTCL [83]. Mycosis fungoides (MF) is the predominant form of PTCL, constituting approximately 60% of all CTCL cases and approximately 50% of primary cutaneous lymphomas [82]. A dominant feature of MF is the presence of UV signature mutations, which contribute to a high tumor mutational burden. It is believed that UV exposure plays a role in the malignant transformation of skin-resident T-cells [84]. Besides, MF exhibits a complex genomic landscape characterized by frequent mutations in various genes involved in different cellular processes. These include genes associated with TCR signaling (PLCG1, CARD11, CD28, RLTPR), epigenetic regulation (TET2, DNMT3A, ARID1A/B), DNA damage response (TP53, POT1, ATM, BRCA1/2), and cell cycle control (CDKN2A/B, TP53) [85]. Moreover, aberrant activation of the NF-kB pathway is commonly observed in MF, primarily due to mutations in genes such as TNFRSF1B, NFKB2, PRKCB, and TNFAIP3 [86]. This activation leads to increased cell proliferation and survival. Furthermore, the JAK-STAT pathway is frequently dysregulated, with copy number gains in STAT3/STAT5B [85]. This dysregulation affects T-cell proliferation, differentiation, and gene regulation. Additionally, disruption of the PI3K/AKT/mTOR pathway, caused by mutations in PIK3CA, RHOA, and VAV1, further impacts T-cell metabolism, growth, and proliferation [27].

Epigenetic changes, including DNA methylation and histone modification, result in the dysregulation of gene expression in MF [87]. Subclonal evolution and intra-tumor heterogeneity are key aspects of MF pathogenesis [88]. These factors contribute to the diversity and complexity of the disease.

### Diagnosis

Early patch/plaque stage MF can clinically mimic benign inflammatory dermatoses such as eczema or psoriasis, which initially presents a diagnostic challenge [89]. However, histopathology in early MF reveals a superficial perivascular and epidermotropic lymphocytic infiltrate. Immunophenotyping further demonstrates the presence of CD4 + small/medium pleomorphic T-cells [89]. To aid in the differentiation of early MF from its mimics, genomic profiling and the identification of mutations in genes such as TET2, DNMT3A, and TP53 can be utilized [85]. These molecular markers provide valuable insights for accurate diagnosis and management of the disease.

As MF progresses to advanced stages, the atypical CD4 + cerebriform lymphocytes become more prominent. In the tumor stage of MF, sheets of atypical lymphocytes can be observed. Additionally, Sezary syndrome, the leukemic variant of MF, is characterized by the presence of clonal circulating Sezary cells [89, 90].

### Treatments

Early-stage MF can be managed using skin-directed therapies, such as topical steroids and phototherapy (UVA/UVB, PUVA). In cases of refractory disease, systemic retinoids or interferons may be employed [85]. For localized plaques and tumors, radiation therapy has proven effective but relapses frequently occur after a few months, and maintenance therapy is mandatory [91].

Conventional chemotherapy, like CHOP (cyclophosphamide, doxorubicin, vincristine, prednisone), yields poor outcomes in advanced MF [92]. However, promising results have been seen in relapsed/refractory cases of MF with the use of novel targeted therapies. These include JAK inhibitors, proteasome inhibitors, HDAC inhibitors, anti-CCR4 antibody, and PD-1/PD-L1 inhibitors [85]. As MF is a complex and chronic disease, it requires a multidisciplinary approach for effective treatment based on disease stage.

## Natural killer T-cell lymphoma (nasal type)

### Pathogenesis

NKTCL-NT is characterized by the malignant proliferation of CD56 + and cytoCD3 + lymphocytes and is known for its aggressive clinical course. This type of lymphoma is more commonly observed in Asian and Latin American populations [93, 94]. The most common sites of occurrence for NKTCL-NT are the nasal cavity, nasopharynx, and palate, followed by the oropharynx, hypopharynx, and tonsils [95].

The pathogenesis of NKTCL-NT involves Epstein-Barr virus (EBV) infection, which act as predisposing risk factors for the disease [96]. In EBV-infected NK/T cells, expression of latent membrane protein 1 (LMP1) and LMP2A is observed. LMP1, which mimics CD40, continuously activates AKT, STAT, JNK, MAPK, and NF-κB pathways. This activation inhibits apoptosis, promotes cell cycle progression, and modulates the immune system. Moreover, LMP1 can induce genomic instability by upregulating activation-induced cytidine deaminase. Genomic instability triggered by EBV infection further leads to somatic mutations in oncogenes and tumor suppressor genes, contributing to the development of EBV-associated NK and T-cell lymphomas [97]. On the other hand, LMP2A mimics the B cell receptor, leading to sustained activation of AKT, Syk, β-catenin, and protein kinase C. Consequently, this sustained activation promotes cell proliferation while inhibiting differentiation. Collectively, these mechanisms contribute to the pathogenesis of EBV-associated NK/T cell lymphoma [97].

The JAK/STAT pathway also plays a significant role for the development of NKTCL-NT. Mutations in the *STAT3* gene are commonly observed in NKTCL-NT [98]. Activation of *STAT3* is significantly correlated with the expression of programmed cell death-ligand 1 (PD-L1), suggesting that STAT3 activation leads to increased PD-L1 expression, promoting immune evasion by the tumor [99]. These findings suggest that immunotherapy targeting the programmed cell death 1 (PD-1)/PD-L1 checkpoint holds promise as a novel therapeutic option. In addition to the JAK/STAT pathway, other potential therapeutic targets in NKTCL-NT include Aurora kinase, MYC, NF-κB, FOXO3, deletion of chromosome 6q21-25, and promoter hypermethylation [100].

### Diagnosis

Common primary symptoms of NKTCL-NT include nasal obstruction, nasal discharge, and nasal bleeding caused by nasal masses [95]. The occurrence of B symptoms is important in assessing NKT [95]. In the pre-treatment evaluation of NKTCL-NT, routine physical examinations, blood tests, biochemical examinations, enhanced whole-body CT scans, enhanced MRI, and endoscopy can be utilized. PET-CT is useful for staging, as lymphomas are known to have high avidity for 18-fluorodeoxyglucose [100]. Additionally, quantification of circulating EBV DNA serves as an accurate biomarker for assessing tumor load [100]. The typical immunophenotype of NKTCL-NT is determined based on pathological histology and immunohistochemistry. It is characterized by the absence of CD20, presence of CD3, lack of CD5, expression of CD56, high Ki-67 proliferation index, and increased levels of cytotoxic molecules such as granzyme B, perforin, and TIA-1 [100].

### Treatments

Stage I NKTCL-NT patients without risk factors (age < 60 years, ECOG score 0–1, normal LDH, no extensive local invasion) can achieve favorable outcomes with radiotherapy alone [101]. On the other hand, stage I patients with risk factors and stage II patients are typically treated with a combination of radiotherapy and chemotherapy as the standard of care [102]. In stages I-II, the success of early NKTCL-NT treatment depends on the radiotherapy field and dose, which are closely associated with local control rates and prognosis [102].

L-menthanate-based chemotherapy regimens have shown the highest effectiveness in systemic treatment for NKTCL-NT [103]. One of these regimens is the SMILE regimen (dexamethasone, methotrexate, ifosfamide, L-asparaginase, and etoposide), which has demonstrated significant efficacy in primary stage III/IV and refractory relapsed cases [104]. Despite the improved response rates with L-menthanate-based chemotherapy, relapse still occurs in approximately 50% of patients with disseminated disease [100]. Targeted therapy, immunotherapy or transplantation may be options for patients with advanced, and relapsed/refractory NKTCL-NT.

The anti-PD-1 inhibitor sintilimab has shown unique efficacy in refractory relapsed NKTCL-NT, with preliminary results indicating an overall response rate of 67.9%, a complete response rate of 7.1%, and a 1-year overall survival (OS) rate of 82.1% [53]. Preliminary results from small-sample studies suggest that pembrolizumab may also have good efficacy [105]. Additionally, a phase II study has demonstrated the effectiveness of the histone deacetylase inhibitor chidamide in some patients, making it a potential option for those with refractory relapses [106].

Conventional chemotherapy alone has poor prognosis for relapsed/refractory NKTCL-NT. Although the value of ASCT remains controversial, several retrospective studies have shown that advanced or sensitive relapsed patients can benefit from ASCT after achieving high-quality remission [107–109]. Allogeneic transplantation is currently being explored due to its associated treatment-related risks but may be considered for refractory patients who have relapsed after autologous transplantation [109].

## Primary central nervous system lymphoma

### Pathogenesis

PCNSL is a highly aggressive, rare form of hematolymphoid tumor that occurs in the CNS, recognized as a primary large B-cell lymphoma of immune-privileged sites by the 5th edition of the World Health Organization Classification of Hematolymphoid Tumors [110–112]. PCNSL occur mostly among patients aged between 50 to70 and the median age at diagnosis is 65 [113]. The incidence of PCNSL has steadily increased over the past two decades, with an annual incidence rate of 0.4–0.5 per 100,000 [114–117]. The prognosis for PCNSL is poor, with a median survival of approximately 26 months [118] and the 5-year and 10-year survival rates of 35.2% and 27.5%, respectively [119].

Pathologically, more than 95% of PCNSL cases are diffuse large B cell lymphoma [120, 121]. Gene expression analysis confirmed that non-germinal center B-cell (GCB) is the most common phenotype in PCNSL patients [121–124]. PCNSL cases often carry mutations that lead to activation of the NF-κB pathway, such as activating mutations in MYD88, CDKN2A, TNFAIP3 and CD79*B*, suggesting that activation of the NF-κB pathway is a key driver of lymphangiogenesis in PCNSL[123, 125–136]. Based on the co-occurrence of the *MYD88*^*L265P*^ and *CD79B* mutations, PCNSL is genetically of the MCD/C5 subtype [123, 125–134]. Common genomic and transcriptional hallmarks of PCNSL also include numerous BCR pathway related gene mutations [137–143], TLR pathway related gene mutations[135, 138, 139, 141–145], chromosomal translocations [146–149], aberrant somatic hypermutation [146, 150, 151].

### Diagnosis

Patients with PCNSL typically emerge within weeks with neurological symptoms, such as focal neurological impairments (56–70%), altered mental state and behavior (32–43%), signs of raised intracranial pressure (headache, nausea, vomiting, optic papilledema; 32–33%), and seizures (11–14%) [152, 153]. On medical imaging, PCNSL usually appears as a uniformly enhancing mass, most commonly as a single brain lesion (66%), with a supratentorial position (87%) and frontoparietal lobe involvement (39%). Less frequently implicated are the eyes (15–25%), CSF (7–42%), and spinal cord (15–25%) [152]. To systematically assess the extent of disease involvement, the International PCNSL Collaborative Group suggests baseline staging, which includes MRI of the brain and spine, ophthalmologic evaluation, and CSF analysis [5]. In addition, a PET/CT and a bone marrow biopsy should be performed to assess whether PCNSL involves the non-central nervous system. The primary method for diagnosing PCNSL is a stereotactic biopsy. If there is a lot of damage to the eye or there are tumor cells in the CSF, a vitrectomy or CSF cytology may help confirm the diagnosis [152].

### Treatments

High-dose methotrexate (HD-MTX) is the basis for the treatment of PCNSL [154–163]. Current major controversies in the treatment of PCNSL include the value and timing of surgery, the optimum chemotherapy regimen, the application of whole brain radiotherapy (WBRT), and the requirement for intrathecal chemotherapy [152]. Due to the high surgical risk posed by the broad and diffuse infiltrative growth of PCNSL, stereotactic biopsy is often employed to confirm the diagnosis. Surgical resection may also increase the risk of irreversible neurological damage [154].

Since the early 1980s, WBRT has been utilized to treat newly diagnosed PCNSL. When combined with HD-MTX, WBRT improved chemotherapeutic response and prolonged PCNSL survival [164–168]. Nonetheless, neurotoxicity has emerged as a significant factor influencing the quality of patient survival [169, 170]. Patients who received WBRT had considerably longer progression-free survival (PFS) than those who did not, but there was no significant improvement in the overall survival (OS) [169]. Clinical specialists are incredibly cautious when administering WBRT to PCNSL patients, especially to the elderlies, owing to the treatment's poor survival and significant neurotoxicity [169]. More alternative therapeutic strategies, including reduced-dose WBRT and local irradiation to the lesion to decrease neurotoxicity, are being evaluated in clinical trials in patients with PCNSL. Rituximab, a monoclonal antibody against B-cell surface antigen CD20, has been shown to enhance the clinical outcomes of PCNSL patients significantly [131, 163, 171–177]. Rituximab is currently used as an induction regimen in PCNSL with common regimens such as R-MVP (rituximab, methotrexate, procarbazine, and vincristine), R-MT (rituximab, HD-MTX, and temozolomide), Matrix (HD-MTX, cytarabine, thiotepa, and rituximab), or R-MVBP (rituximab, methotrexate, etoposide, carmustine, dexamethasone). Choosing the most appropriate chemotherapy regimen for PCNSL patients is a pressing challenge in clinical work. Lastly, there is no agreement on whether chemotherapy should be applied intracerebroventricularly. Even though intrathecal chemotherapeutic agents may prolong exposure to cytotoxic drugs in the CSF, they can also increase neurotoxicity [178].

Several novel treatments have shown efficacy and overall good tolerance in PCNSL patients, such as ASCT [179–188], BTK inhibitors [56, 189–198] and chimeric antigen receptor T-cells (CAR-T) [199–201].

## Prognostic markers for PCNSL

In the past few decades, the prognosis of PCNSL has significantly improved due to the widespread use of HD-MTX chemotherapy and consolidation therapy. However, relapse remains common, with a 5-year survival rate of only 30% to 40% [114, 154]. Currently, common prognostic markers for PCNSL include basic characteristics, subtypes, imaging findings, prognosis scoring systems, clinical laboratory results, and biomolecules.

### Utilization of basic patient characteristics as prognostic markers

#### Basic characteristics of PCNSL patients

According to a study comprising 466 PCNSL patients from 62 Japanese medical institutions, age > 60 years and the Eastern Cooperative Oncology Group Performance Status (ECOG PS) score > 2 were found strongly related to poor prognosis in PCNSL patients [202]. In a second study, Niparuck et al. additionally confirmed that ECOG PS score > 1 may function as an independent predictor of OS in multivariate analysis [203]. Furthermore, type B symptoms, multifocal lesions, meningeal spread, and higher lactate dehydrogenase (LDH) levels were linked to a worse prognosis [202].

#### Tumor localization

Patients with PCNSL have bad undesirable prognosis if the tumors are located in the deep brain, including the periventricular zone, basal ganglia, corpus callosum, brainstem and/or cerebellum [204–208]. Multivariate analysis of 101 newly diagnosed patients with PCNSL showed that deep brain lesions were an independent risk factor for PFS [204]. Another retrospective analysis of the clinical data of 89 patients with intracranial PCNSL by Ouyang et al. in 2020 showed that deep structural invasion was the independent risk factor for intracranial PCNSL [205]. Furthermore, patients with deep brain involvement have a higher risk of mortality in the first few months after diagnosis [208].

#### Mini-mental state examination

Mini-Mental State Examination (MMSE) is a tool for screening neurocognitive disorders [209–212]. In low-grade and high-grade gliomas, the MMSE score was an independent predictor of PFS and OS [213, 214]. A multicenter, phase III, and randomized trial examined the predictive value of the MMSE in 199 patients with PCNSL. All study subjects were adults with an ECOG PS score of 0 to 3, normal immune function, and CD20 positivity. One hundred and fifty-three patients out of 199 had MMSE scores at baseline. The MMSE score functioned as an independent predictor for OS and PFS in multivariate analysis. To summarize, the MMSE score is not only helpful in assessing the prognosis of patients with PCNSL, but it also straightforward and easy to use, making it useful in clinical practice [215].

### Utilization of PCNSL cell of origin-based subtypes as prognostic markers

The classical PCNSL subtype by immunohistochemistry is based on the Hans algorithm, which is sorted by CD10, Bcl-6, and MUM-1 expression. Double expressor lymphoma has been utilized to classify PCNSL subtypes in recent years.

**Fig. 2:**
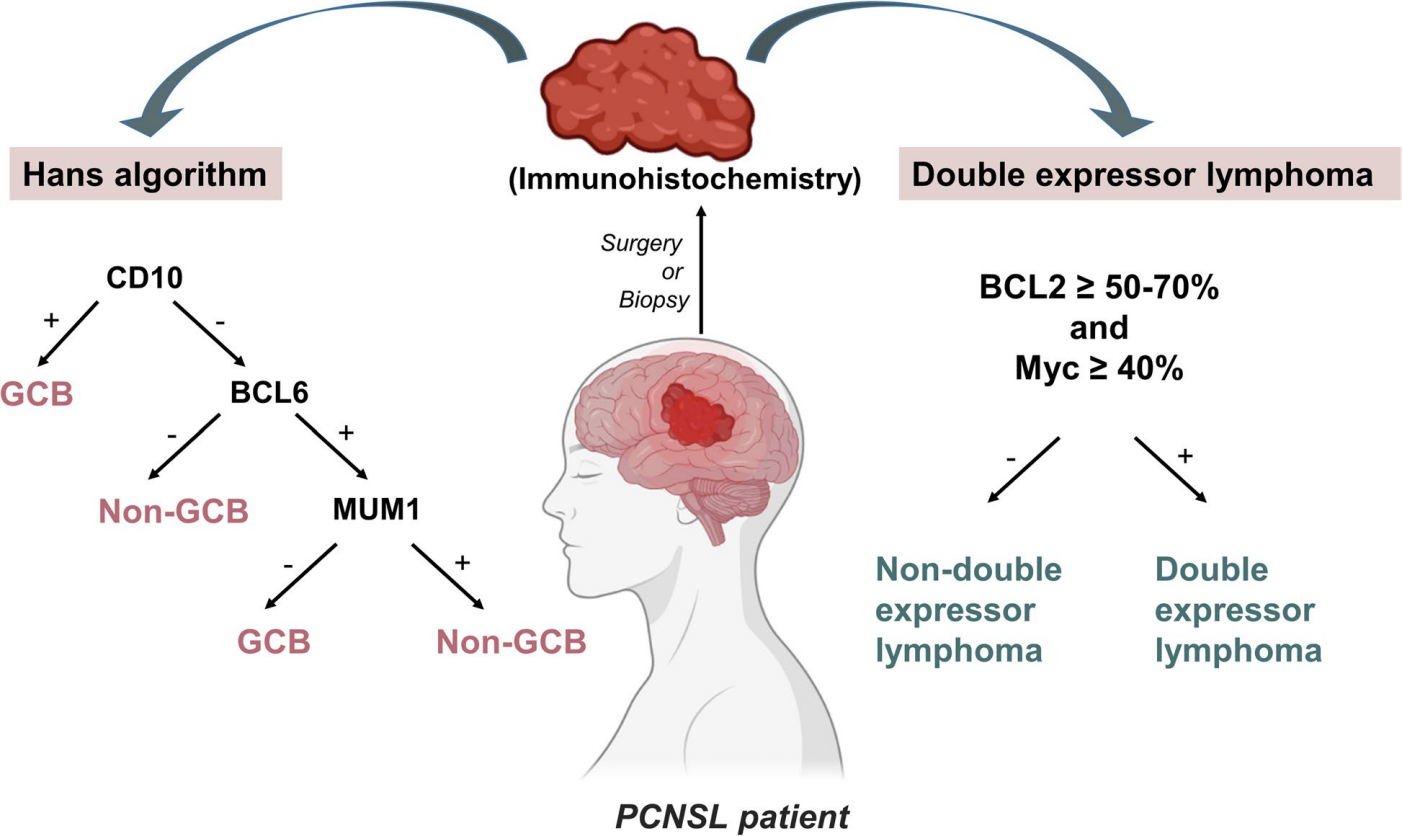
The classification criteria for the two subtypes of PCNSL. Left: Hans algorithm; Right: Double expressor lymphoma; + : Positive expression; -: Negative expression. Abbreviations: GCB, Germinal center B-cell; PCNSL, Primary central nervous system lymphoma

Figure 2 displays the classification criteria for the two subtypes of PCNSL.

#### Hans algorithm

DLBLC can be classified as GCB and non-GCB subtype according to Hans algorithm. The GCB subtype is associated with better prognosis in DLBCL [114]. Non-GCB was the most common phenotype in PCNSL patients, accounting for 65.7–96.3% of cases [121, 123, 130, 203, 216–219]. Besides, PCNSL was more commonly categorized in the non-GCB subgroup than DLBCL of peripheral nodal origin (*p* = 0.020; 78% *vs.* 62%), which may be primarily attributable to the increased nuclear MUM-1, also known as IRF-4, expression in PCNSL [220].

Hans algorithm may assist in determining the prognosis of PCNSL patients. In 2017, a study analyzed clinical, neuroimaging, and immunohistochemistry data from 41 PCNSL patients, who mostly received methotrexate-based chemotherapy-radiotherapy, to determine the impact of potential prognostic markers on clinical outcomes and the linkage between these markers. The GCB subtype was associated with a trend toward improved survival. However, neither OS nor PFS were statistically significant (*p* = 0.139 and *p* = 0.167, respectively) [218]. Another study included 43 patients with PCNSL, all receiving HD-MTX-based regimens, WBRT, or both. The OS of PCNSL was favorably linked with the GCB subtype [203]. Besides, a study investigated specimens and clinical data from 24 patients with biopsy-proven PCNSL and found that after a median follow-up of 15 months, only 39% of patients with non-GCB type PCNSL were alive, whereas all GCB type patients were alive. The median survival time for non-GCB patients was 11 months, but all GCB patients were still alive after a median follow-up period of 22 months [221]. Mechanistically, aberrant expression of BCL2 [203] or phosphorylation of the STAT3 protein [222] may contribute to the poor prognosis of PCNSL patients with non-GCB subtypes.

Although some investigations have shown no significant survival differences between the two categories of GCB and non-GCB [216, 219, 223], current studies tend to suggest that the prognosis for PCNSL patients with the GCB subtype of DLBCL is favorable.

#### Double expressor lymphoma

The identification of concurrent *MYC* and *BCL2* (or *BCL6*) deregulation, whether at a genomic or protein level, has opened a new era of investigation within the most common subtype of PCNSL. Double-hit lymphoma (DHL), defined as a dual rearrangement of *MYC* and *BCL2* and/or *BCL6* genes [224–227]. Double-expressor lymphoma (DEL), defined as overexpression of c-MYC and BCL2 proteins not related to underlying chromosomal rearrangements [224, 228–231]. Both DHL and DEL are associated with a more aggressive clinical course and a worse prognosis for DLBCL patients [232]. Compared to DHL, DEL is more common in patients with PCNSL[130, 233, 234]. Therefore, we focused on the impact of DEL on the prognosis of PCNSL patients.

In a cohort of 48 individuals with newly diagnosed PCNSL, Hatzl S et al. followed 48 patients with newly diagnosed PCNSL for a median of 6.2 year. PCNSL patients with DEL characteristics had a 5-year risk of progression and/or death that was 13 times greater than those without DEL characteristics. Moreover, adding DEL in the International Prognostic Index (IPI) increases the model's prediction accuracy [235]. In 2022, a retrospective analysis was conducted on 82 pathologically proven, CD20-positive, PCNSL patients aged 71 or older who received therapeutic intervention in Japan. DEL was present in 43/82 (52.4%) cases. Multivariate analysis of the median OS revealed that DEL was the pathogenic risk factor [hazard ratio (HR) = 3.163, *p* = 0.004] [236]. A meta-analysis also confirmed that DEL was significantly associated with short median OS (HR = 1.23, *p* = 0.001) [237].

### Imaging data as a prognostic indicator for PCNSL

#### Temporalis muscle thickness and L3 lumbar skeletal muscle index

Two muscle mass markers, temporalis muscle thickness (TMT) and L3 lumbar-skeletal muscle index (L3-SMI), were revealed to be independent predictors of PCNSL outcome. TMT is measured by MRI, which was found to be an independent predictor of OS in a study of 128 patients with primary PCNSL who had cranial MRI data [238]. In another study, 43 PCNSL patients who received first-line HD-MTX-based chemotherapy underwent brain MRI, and whole-body CT scans within 30 days of beginning treatment. Patients with low TMT levels had significantly worse PFS (HR = 4.40, *p* = 0.003) and OS (HR = 4.93, *p* = 0.002) than those with high TMT values [239].

The L3-SMI was calculated by first measuring the surface area of the abdominal and paraspinal muscles contained in the axial profile acquired at the third lumbar vertebra and then dividing the surface area by the square of the patient's height. According to the COX multivariate analysis in the preceding study [239], patients with low L3-SMI values had significantly shorter PFS (HR = 4.40, *p* = 0.003) and OS (HR = 3.16, *p* = 0.034) than those with high L3-SMI values.

#### Apparent diffusion coefficient

There are signs that a higher tumor cell density in diagnostic samples of PCNSL may have important prognostic effects. Because cellular density is negatively correlated with apparent diffusion coefficient (ADC) measurements on diffusion-weighted MRI (DWI), ADC values may predict the clinical prognosis of PCNSL patients [240]. The results suggest that lower ADC is associated with shorter PFS [240–242] or OS [240, 241, 243].

ADC values also correlated with the efficacy of HD-MTX-based chemotherapy regimens. A retrospective study of 28 patients treated with HD-MTX-based chemotherapy shows that there was a substantial between complete response (CR) and non-CR in terms of ADC_mean_ and ADC_5%_ percent. In addition, ADC_5%_ percent beat ADC_mean_, as the area under the ROC curve (AUC) was greater for ADC_5%_ compared to ADC_mean_ (0.983 *vs.* 0.822) [242].

In summary, ADC values predicts PFS, OS, and the efficacy of HD-MTX in PCNSL patients.

#### Fluorodeoxyglucose-PET

Due to the high density of PCNSL tumor cells, quick glucose metabolism and high FDG content in the tumor, PCNSL demonstrates significant FDG uptake and can be diagnosed with an excessive degree of sensitivity using FDG-PET [244, 245]. FDG-PET can differentiate PCNSL from other forms of brain cancer [246–249]. In addition, FDG-PET may be more sensitive than conventional physical staging in the diagnosis of PCNSL and may detect the presence of additional concomitant systemic disorders [245, 250, 251]. Thus, FDG-PET is a non-invasive approach that may give verified prospective prognostic information for patients with PCNSL.

Kawai et al. performed FDG-PET in 17 patients with newly diagnosed PCNSL before treatment. FDG uptake was assessed by showing the standardized uptake value (SUV) of the tumor, showing the maximum uptake (SUVmax). This study showed that patients in the low and moderate uptake group (SUVmax < 12) had significantly better OS and PFS than those in the high uptake group (SUVmax ≥ 12), and therefore pretreatment FDG uptake could be used as a prognostic indicator for PCNSL [252]. Of note, Tateishi et al*.* found that NF-kB pathway activated RelA/p65-hexokinase 2, a rate limiting enzyme for glycolytic pathway [125]. Since most PCNSL harbors mutations in the MYD88 and CD79B, an upstream gene of the NF-kB canonical pathway, these mutations may contribute high uptake of FDG in PCNSL.

### Prognosis scoring systems

**Fig. 3:**
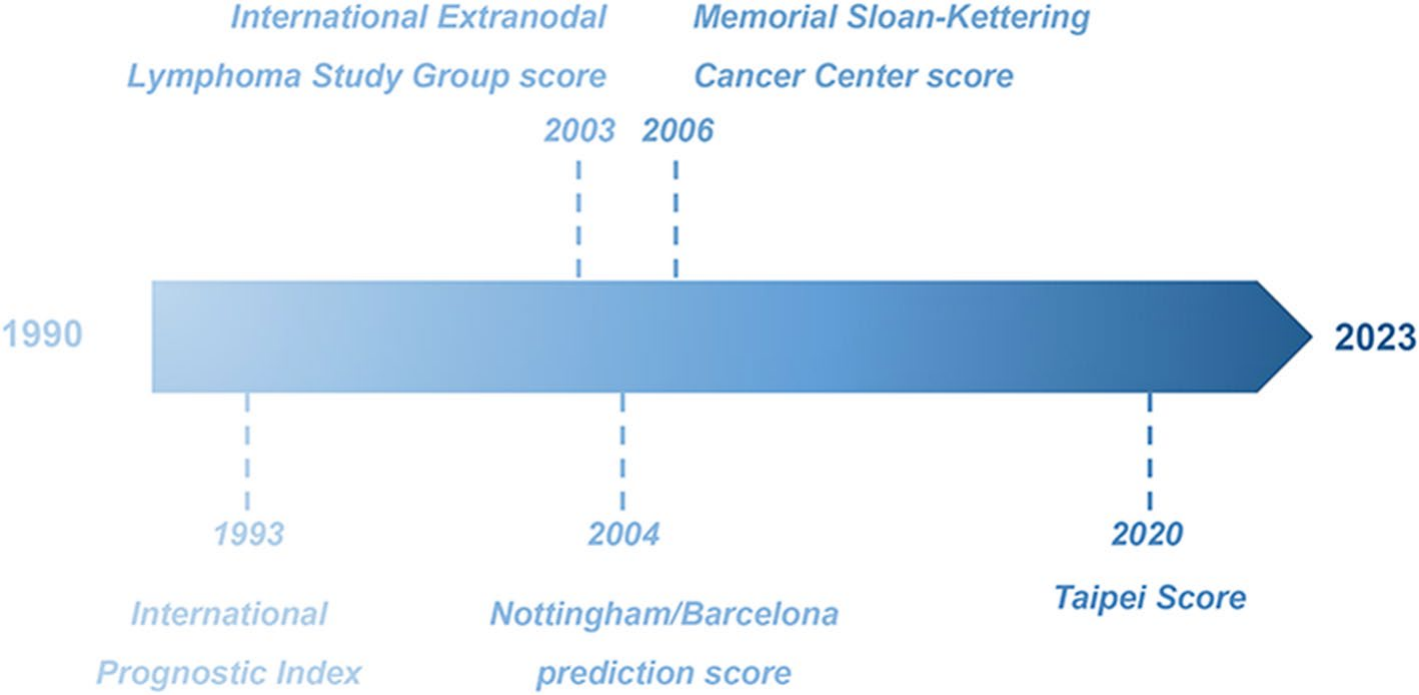
The schematic plot of the progress for PCNSL prognostic scoring system. Abbreviations: PCNSL, Primary central nervous system lymphoma

For decades, five prognostic indexes have been proposed to stratify the clinical evolution of PCNSL (Fig. 3). Table 2 displays the detailed variables, hazard stratification, cancer types applied for the first time and disadvantages for these five prognosic scoring systems.

**Table 2:** Summary of five prognostic scoring systems

	**The International Prognostic Index** **(IPI)**		**The International Extranodal Lymphoma Study Group score** **(IELSG)**		**The Nottingham/Barcelona prediction score**		**The Memorial Sloan-Kettering Cancer Center score** **(MSKCC)**		**The Taipei Score**	
**Variables**	**Favorable factors** **(0 points)**	**Negative factors** **(1 point)**	**Favorable factors** **(0 points)**	**Negative factors** **(1 point)**	**Favorable factors** **(0 points)**	**Negative factors** **(1 point)**	**Favorable factors**	**Negative factors**	**Favorable factors** **(0 points)**	**Negative factors** **(1 point)**
	① Age < 60 ② ECGO score ≤ 1 ③ Normal LDH level ④ Ann Arbor stage I-II ⑤ Number of extranodal locations of illness ≤ 1	① Age ≥ 60 ② ECGO score > 1 ③ Elevated LDH levels ④ Ann Arbor stage III-IV ⑤ Number of extranodal locations of illness > 1	① Age ≤ 60 years ② ECGO score ≤ 1 ③ Normal LDH level ④ Normal protein level in CSF ⑤ No deep CNS involvement	① Age > 60 years ② ECGO score > 1 ③ Elevated LDH levels ④ Elevated protein levels in CSF ⑤ Deep CNS involvement	① Age < 60 ② ECGO score ≤ 1 ③ No multifocal and/or meningeal disease	① Age ≥ 60 ② ECGO score < 2 ③ Multifocal and/or meningeal disease	① Age < 50 ② KPS ≥ 70	① Age ≥ 50 ② KPS < 70	① Age < 80 ② ECGO score < 2 ③ No deep brain lesions	① Age ≥ 80 ② ECGO score ≥ 2 ③ Deep brain lesions
**Hazard stratification**	Low-risk group: 0–1 Intermediate-low risk group: 2 Intermediate-high risk group: 3 High-risk group: 4–5		Low-risk group: 0–1 Medium risk group: 2–3 High-risk group: 4–5		Each point scored corresponds to a level		Low-risk group: Age < 50 Medium risk group: age ≥ 50 and KPS ≥ 70 High-risk group: age ≥ 50 and KPS < 70		Each point scored corresponds to a level	
**Cancer types applied for the first time**	Non-Hodgkin's lymphoma		Primary central nervous system lymphoma		Primary central nervous system lymphoma		Primary central nervous system lymphoma		Primary central nervous system lymphoma	
**Disadvantages**	① Only the first two variables are clearly relevant to PCNSL; ② The Ann Arbor stage of PCNSL is controversial		① Relatively small Number of patients (*N* = 105); Short follow-up time (median, 24 months); ② Patients do not typically undergo a lumbar puncture before starting treatment, resulting in a substantial proportion of patients lacking CSF protein levels for IELSG scoring		① Relatively small Number of patients (*N* = 77); ② No uniform chemotherapy regimen and based on old-fashioned chemotherapeutic regimens; ③ Very little information is available on the long-term survival data		① This study has an issue of selection bias since all patients are from the same institution; ② Because there are only two variables, some clinical studies cannot verify their validity		① Retrospective research; Single Centre Research; The patient population is primarily Asian; ② Numerous patients lack sufficient cytogenetic and molecular information for subtyping	

*ECGO* Eastern Cooperative Oncology Group, *LDH* Lactate dehydrogenase, *KPS* Karnofsky performance score

#### International prognostic index

The IPI is a broadly acknowledged prognostic score that may be used to differentiate between various risk categories of patients with DLBCL [253]. Twenty-five immunocompetent adult patients with PCNSL were treated in an early experiment with five cycles of HD-MTX-based chemotherapy followed by cerebral irradiation. The 2-year OS was 0% for patients > 60 years old with an IPI of 3 or more, compared to 88% for patients ≤ 60 years old with an IPI of 4 or less. The prognostic value of IPI in PCNSL was proven in this study [254].

#### International extranodal lymphoma study group score

In 2003 Ferreri et al. proposed the International Extranodal Lymphoma Study Group (IELSG), a scoring system based on clinical data of 378 PCNSL patients from 23 cancer centers in 5 countries from 1980 to 1999. The mean follow-up period was 24 months. It was found that age > 60 years, ECOG PS score > 1, elevated LDH levels, elevated CSF protein concentrations, and deep brain involvement could be independent prognostic markers for PCNSL. Based on the above findings, 105 evaluable patients were analyzed, and an IELSG prognostic model was developed, classifying adverse prognostic markers of 0–1, 2–3, and 4–5 as low, intermediate, and high-risk groups with 2-year OS rates of 80%, 48%, and 15%, respectively [206].

The prognosis model's accuracy can be increased by using the IELSG score in combination with additional prognostic parameters. A stronger predictive relevance can be seen, for instance, when the IELSG score is paired with the expression of programmed cell death ligand-1 (PD-1) on tumor-associated macrophages (IELSG-M). For OS, the areas under the receiver operating characteristic curves of IELSG-M were 0.844, which was higher than the IELSG model (0.580) [255].

#### Nottingham/Barcelona prediction score

The Nottingham/Barcelona prediction score includes three adverse prognostic variables, each with a value of 1. OS was negatively correlated with the Nottingham/Barcelona prediction score. The median survival for the 77 PCNSL patients included in the study was 55, 41, 32, and 1 month, with scores of 0, 1, 2, and 3, respectively [256].

#### Memorial Sloan-Kettering cancer center score

Age and karnofsky performance score (KPS) were the only two variables included in the Memorial Sloan-Kettering Cancer Center score (MSKCC score), and they were used to stratify participants into low-, intermediate-, and high-risk groups. The OS in PCNSL patients was negatively correlated with the MSKCC prognostic model score [207, 257, 258]. The largest study had 338 consecutive individuals with newly diagnosed PCNSL. The median OS for the low-, intermediate- and high-risk groups were 8.5, 3.2, and 1.1 years (*p* < 0.001), respectively. The median failure-free survival for the low-, intermediate- and high-risk groups were 2, 1.8, and 0.6 years (*p* < 0.001) [257]. Notably, one study found no significant difference in OS between the low- and intermediate-risk groups based on the MSKCC score [258]. Additional prognostic variables, such as lactate dehydrogenase/lymphocyte ratio (LLR) [258] and hemoglobin (Hb) [259], should be added to the MSKCC model to improve it further.

#### Taipei score

The researchers discovered that the IELSG, Nottingham/Barcelona, and MSKCC models are not sufficiently satisfactory for differentiating PFS or OS in patients with PCNSL. In order to construct a more accurate prognostic model, the researchers recruited 101 newly diagnosed PCNSL patients. Age ≥ 80 years, ECOG PS score ≥ 2, and deep brain lesions were identified as independent adverse prognostic markers for PFS by multivariate analysis. Researchers scored one point for each adverse prognostic factor and developed a new predictive model, the Taipei score, with four different risk categories (scores 0–3). In the training cohort, the Taipei score distinguished between PFS and OS significantly, and the score was verified in an external validation cohort. The Taipei score is therefore expected to provide the classification of disease risk for PCNSL and improve clinical decision-making [204].

### Utilization of routine hematological indicators as prognostic indicators

Pre-treatment hematology is a routine test for all patients and is a convenient way to predict the prognosis of PCNSL. Hematologic indicators also can be used in combination with prognosis scoring systems to improve predicted accuracy. Table 3 displays the findings of studies utilizing conventional hematological clinical markers as prognostic indicators.

**Table 3:** Summary of studies investigating haematological clinical markers as prognostic factors

PMID	Factor	Retrospective or Prospective	Number of patients/centers involved	Treatment	Median Age (range), year	PFS	OS
26918738 [260]	ALC	Retrospective	81/1	HD-MTX	59 (33–79)	HR = 3.1, *p* = 0.001	HR = 2.83, *p* = 0.008
29088839 [261]	NLR	Retrospective	62/2	HD-MTX	63 (21–81)	HR = 2.28, *p* = 0.073	HR = 2.36, *p* = 0.102
34422649 [258]	NLR	Retrospective	248/6	/	59 (21–86)	/	HR = 1.634, *p* = 0.023
33996552 [262]	NLR	Retrospective	60/1	HD-MTX	57 (18–79)	HR = 10.54, *p* = 0.034	/
34422649 [258]	LLR	Retrospective	248/6	/	59 (21–86)	/	HR = 1.792, *p* = 0.015
30867243 [259]	Hemoglobin (anemia)	Retrospective	91/1	/	65 (58–73)	/	Cohort A: HR = 2.7, *p* = 0.001; Cohort B: HR = 2.5, *p* = 0.001
33996552 [262]	Hemoglobin (anemia)	Retrospective	60/1	HD-MTX	57 (18–79)	HR = 3.940, *p* = 0.013	/
33996552 [262]	LMR	Retrospective	60/1	HD-MTX	57 (18–79)	/	HR = 24.040, *p* = 0.019
33996552 [262]	SII	Retrospective	60/1	HD-MTX	57 (18–79)	/	HR = 11.174, *p* = 0.002
33996552 [262]	TBIL	Retrospective	60/1	HD-MTX	57 (18–79)	HR = 3.429, *p* = 0.004	HR = 5.245, *p* = 0.002

*ALC* Lymphocyte count, *EFS* Event-free survival, *Hb* Hemoglobin, *HD-MTX* High-dose methotrexate, *LLR* Lactate dehydrogenase/lymphocyte ratio, *LMR* Lymphocyte/monocyte ratio, *NA* Not mentioned, *NLR* Neutrophil/lymphocyte ratio, *OS* Overall survival, *PFS* Progression-free survival

#### Lymphocyte count

Lymphocyte count (ALC) has predictive relevance in non-Hodgkin’s lymphoma [260, 263–265]. In 2016, Korean researchers first discovered that pretreatment ALC also could be an independent prognostic marker in PCNSL patients. They analyzed 81 PCNSL patients treated with HD-MTX and developed a new predictive model based on ECOG PS score > 1, age > 50 years, and the existence of decreased ALC, assigning 1 point to each factor and categorizing patients into three risk groups: low (0–1), intermediate (2), and high (3). Patients in the low, moderate, and high-risk categories had 5-year survival rates of 74.3%, 21.7%, and 12.5%, respectively [260]. Because of the convenience and low cost of detecting ALC, this model could be utilized as an objective and reliable prognostic tool for PCNSL. Notably, the predictive importance of ALC and this model needs be confirmed in a larger number of samples.

#### Neutrophil/lymphocyte ratio

Tumor cells release cytokines and chemokines to attract immunological and inflammatory cells, which stimulate tumor growth and survival [266–276]. A high neutrophil count may be a marker of inflammation, while a low lymphocyte count may indicate a lack of host immunity [277]. Hence, a high neutrophil/lymphocyte ratio (NLR) before therapy may therefore be one of the negative prognostic variables. Recently, high pre-treatment NLR has been proven to be an independent marker of poor prognosis in DLBCL [278, 279].

For PCNSL, high NLR was an independent prognostic factor [258, 261, 262]. High NLR was significantly associated with a worse PFS [261] and OS [258, 261] for PCNSLs by univariate analysis. Due to the strong lympho-toxic effects of steroids, the use of steroids prior to chemotherapy in PCNSL patients may affect NLR [280]. In the recent study, 75 individuals who had received chemoimmunotherapy were included. The study calculated NLR at three-time points: baseline (pre-steroid), pre-chemoimmunotherapy (post-steroid) and post-chemoimmunotherapy. The results suggest that OS was longer with higher pre-chemoimmunotherapy (post-steroid) NLR (dichotomized at NLR ≥ 4.0, HR = 0.42, 95% CI: 0.21–0.83, *p* = 0.01) [280]. It is hypothesized that steroid therapy, when combined with NLR, can successfully calibrate the PCNSL prognostic model and increase the accuracy of NLR in determining patient prognosis.

#### Lactate dehydrogenase/lymphocyte ratio

The lactate dehydrogenase/lymphocyte ratio (LLR) has been shown to be an independent prognostic factor in patients with extranodal natural killer/T-cell lymphoma [281], DLBCL [282, 283] and metastatic renal cell carcinoma [284]. Clinical data from 248 patients with PCNSL diagnosed at six cancer facilities in 4 countries were analyzed from 2004 to 2019 to see if LLR could be used as a promising predictive model for PCNSL. OS was selected as the study's endpoint. According to univariate analysis, LLR values greater than 166.8 were significantly related to a poorer OS. LLR was also shown to be an independent prognostic parameter for poorer OS by multivariate analysis. Notably, there was no significant difference in OS between the low- and intermediate-risk groups according to the MSKCC score; however, LLR could be an independent prognostic indicator for these patients [258].

#### Hemoglobin

Anemic individuals account for 30% to 90% of cancer patients [285–288]; nevertheless, Hb measurement is impacted by potentially confounding factors. The most common confounding variable is the use of corticosteroids by a portion of PCNSL patients, which may influence hemoglobin levels. Additionally, patients with tumors frequently have one or more concurrent anemia-causing causes, such as inflammatory anemia, chronic illness anemia, or bleeding disorders. Hb was an independent prognostic factor for PCNSLs (HR = 3.94, *p* = 0.013) [262]. In 2019, a retrospective study of 182 newly diagnosed PCNSL patients from a single medical center indicated that anemia was significantly associated with poor OS. Notably, combining Hb enhances MSKCC's accuracy in predicting PCNSL outcomes [259].

#### Systemic immune inflammatory index, lymphocyte/monocyte ratio and total bilirubin

There is growing evidence that cancer-related inflammation can promote the growth, invasion, and metastasis of cancer cells [289–299]. As a component of the innate immune system, neutrophils are an indicator of ongoing systemic inflammation. Additionally, neutrophils may contribute to the suppression of lymphocyte function, promote tumor immune escape and facilitate metastasis [300]. Nevertheless, the predictive significance of peripheral blood markers indicative of systemic inflammation and nutritional status in patients with PCNSL is uncertain. Systemic immune inflammatory index (SII) is an index of systemic inflammatory response calculated from platelet count × neutrophil count/lymphocyte count. A retrospective study analyzed 60 patients with HD-MTX-based standard chemotherapy PCNSL diagnosed from 2011 to 2020. Lymphocyte/monocyte ratio (LMR) (HR = 24.040, *p* = 0.019), SII (HR = 11.174, *p* = 0.002) and total bilirubin (TBIL) (HR = 5.245, *p* = 0.002) were independently associated with OS in this multivariate analysis. The C-index of the MSKCC score increased from 0.57 to 0.72 when SII and TBIL were added, indicating that the addition of SII and TBIL improved the ability of the MSKCC score to predict survival in PCNSL patients treated with the HD-MTX regimen [262].

### Biomolecules as prognostic indicators in PCNSL

#### miRNAs

MicroRNAs (miRNAs) are involved in every biological process relevant to cancer, including cell proliferation, differentiation, death, and metabolism [289–299]. Importantly, the biogenesis and activation of miRNAs are faster with longer half-lives compared to mRNA and proteins, which may make miRNAs more suitable for earlier detection [301–310].

One study examined the levels of circulating miRNAs in PCNSL patients and found that miR-151a-5p and miR-151b could significantly differ short-term from long-term survival [311]. Mao et al*.* found that miR-21 was significantly elevated in the serum of PCNSL patients compared to other brain tumors and normal controls. Kaplan–Meier survival curves shown higher expression level of serum miR-21 was tightly associated with a poor prognosis in both test and validation cohorts [312]. In another trial assessing the efficacy of pemetrexed plus rituximab as second-line treatment, higher blood miR-21 levels indicated shorter survival, with a PFS of 5.7 months compared to 9.0 months when serum miR-21 levels were lower [313]. miR-30d, miR-93, miR-181b [314], miR-101, miR-548b, miR-554, and miR-1202 [315] have also been reported to be promising as useful prognostic markers for PCNSL. Eight hundred and forty-seven miRNAs expressed in 27 PCNSL specimens were analyzed using microRNA microarrays by Takashima et al. Multivariate analysis revealed that the combination consisting of miR-30d, miR-93 and miR-181b was an independent factor for poor OS in PCNSL [314]. In addition, Takashima et al*.* detected 847 miRNAs in 40 PCNSL patients using microRNA microarrays, containing 334 miRNAs associated with cancer immune-related genes (associated with regulation of type 1/2 T-helper (Th) cell status, T-reg cell status and immune checkpoints status, respectively), using four of these representative miRNAs (miR- 101, miR-548b, miR-554, and miR-1202) combined with patient clinical information to obtain a prediction formula, and patients in the low group had better OS [315].

#### snRNAs

Small nuclear RNAs (snRNAs) are a subtype of short-stranded non-coding RNA [316–318]. Existing research on the prognostic and diagnostic significance of snRNAs is still limited. Given that circulating U2 small ribonucleic acid fragments (RNU2-1f) serve as novel blood biomarkers for pancreatic, colorectal, and lung malignancies, the function of RNU2-1f in the CSF of PCNSL patients was investigated [319]. Researchers collected sequential CSF samples from nine PCNSL patients and then used real-time PCR to evaluate RNU2-1f levels. The results indicated that CSF RNU2-1f expression was positively linked with disease development based on serial measurements of RNU2-1f from nine patients with varying disease stages. In addition, CSF RNU2-1f levels appeared to correspond with MRI-measured tumor volume. The results presented above demonstrate that the level of RNU2-1f in CSF is a viable biomarker for determining the prognosis of PCNSL [319].

#### MYC

*MYC* (also called c-MYC in protein level) is one of the most prominent prognostic factors in PCNSL and can function at three levels: RNA, DNA, and protein. In a retrospective analysis, Gomes Candido Reis D et al. identified overexpression of *MYC* as a poor prognostic indicator of PCNSL [47]. RNA was isolated from 35 formalin-fixed and paraffin-embedded (FFPE) tissue samples. Following this, quantitative reverse transcription-PCR was performed for *MYC*. Relative gene expression of *MYC* ≥ 0.201 was linked with worse OS (HR = 6.117, *p* = 0.003) and worse PFS (HR = 3.960, *p* = 0.016). Another study found significant differences between the Kaplan–Meier curves in the mutant and wild-type groups, suggesting that somatic mutations in MYC (HR = 0.305, *p* = 0.0012) at the DNA level were associated with better overall survival (OS). These findings indicate that somatic mutations occurring specifically in the *MYC* are potentially important diagnostic and prognostic markers for PCNSL tumorigenesis and patient survival [320]. Overexpression of c-MYC [218, 235, 321] in protein level is also widely recognized to be associated with poor prognosis in PCNSL. To comprehensively assess the predictive role of c-MYC protein expression in PCNSL, Ge et al. conducted a meta-analysis [237]. Thirty-one studies involving 1739 patients were included in this meta-analysis. C-MYC expression was significantly associated with median OS and PFS. Subgroup analysis revealed that c-MYC protein positive remained a significant predictor of short median OS in studies with 45 participants, no WBRT, a quality scale score over 6, and a positivity threshold set at 40% stratum.

#### BCL2 and BCL6

The prognostic role of BCL2 and BCL6 in PCNSL remains controversial. Overexpression of BCL2 [235, 321], and/or BCL6 [218, 321] is generally believed to be associated with a poor prognosis in PCNSL. However, contradictory findings have been reported regarding the predictive value of BCL2 [322] and/or BCL6 [219, 322] in predicting survival in PCNSL patients.

The disparate outcomes of the research above may be attributable to the small sample sizes of the trials and the variety of patients' treatment regimens. The meta-analysis mentioned above also comprehensively assessed the predictive role of BCL2 and BCL6 protein expression in PCNSL [237]. BCL6 protein positivity is associated with a favorable prognosis. There was no significant correlation between BCL2 expression and OS or PFS, but BCL2 and c-MYC co-expression were significantly associated with short median OS. As most of these included papers are retrospective studies, the prognostic effect of BCL2 in PCNSL needs further validation.

#### CD79B

Recurrent mutations in *CD79B* are characteristic of PCNSL, and 69–83% of PCNSL patients were found to have recurrent *CD79B* mutations by sequencing [57, 58]. Recurrent *CD79B* mutations were found in 69–83% of PCNS L patients. The relationship between *CD79B* and PCNSL prognosis is not yet clear. According to Zhou Y et al., patients with lymphoma who harbored the *CD79B* mutation had significantly worse PFS than patients with wild-type *CD79B* [58]. Another study presented the opposite result. Another study with Hispanic PCNSL patients revealed the opposite findings, demonstrating that *CD79B* mutations were associated with improved 2-year PFS [323].

#### MYD88

*MYD88*^*L265P*^, is an important oncogene for lymphoma [324–327]. With the advancement of high-throughput molecular technologies, it has been found that mutations in the *MYD88*^*L265P*^ gene are present in 55–88% of patients with PCNSL [132, 328–331]. Moreover, the protein expression of MYD88 was significantly elevated in PCNSL patients in comparison to individuals with lymphadenitis (70.18% *vs.* 15%) [58].

Hattori K et al. demonstrate for the first time that *MYD88*^*L265P*^ mutation is independently associated with shorter OS and PFS in PCNSL [329]. *MYD88*^*L265P*^ mutation is more prevalent in patients over 65 years old. The Kaplan–Meier analysis revealed that *MYD88*^*L265P*^ mutation predicted shorter OS (11.5 months *vs.* 56.2 months, *p* < 0.04) in patients older than 65 years [330]. Besides, Zhou Y et al. investigated tissue samples from 57 PCNSL patients using immunohistochemistry and discovered that a high level of MYD88 expression was an independent predictor of OS (HR = 0.143, *p* = 0.004) [58]. PCNSL patients with high MYD88 expression had a shorter OS than those with low expression (8 months *vs.* 31 months, *p* = 2.0 × 10^−6^).

However, a study suggested that *MYD88*^*L265P*^ mutation is a favorable prognostic factor for PCNSL. *MYD88*^*L265P*^ mutation status was available in 41 PCNSL patients with non-GCB subtypes, 36 (88%) of whom were mutants. The *MYD88*^*L265P*^ mutation was linked to better survival in the multivariable model (HR = 0.277; *p* = 0.023) [328].

#### ATP binding cassette subfamily B member 1

*ATP Binding Cassette Subfamily B Member 1 (ABCB1*), one of the key ABC transporters of the blood–brain barrier (BBB), can be classified into two genotypes with T (genotypes CT and TT) and without T (genotype CC) [332–334]. The rs1045642 is the most common of the *ABCB1* gene polymorphisms [335]. It has been reported that the CC genotype of *ABCB1* rs1045642 is related to MTX-induced mucositis [336] and poorer event-free survival (EFS) [337] in hematological tumors. Wu et al. conducted a prospective study of 91 patients with PCNSL enrolled at Huashan Hospital from 2006–2015. Multivariate analysis showed that *ABCB1* rs1045642 was an independent risk factor for PFS and was associated with a higher risk of progression, suggesting that assessing the genetic variability of patients provides another possible method to assess the prognosis of PCNSL [335].

#### Ki-67

Ki-67 expression levels indicate the level of cell proliferation. Ki-67 (90% cutoff) was associated with shorter OS (*p* = 0.037) and PFS (*p* = 0.039) in a cohort of 89 PCNSL cases. However, in the multivariate analysis, Ki-67 failed to predict prognosis [322]. In another study that included 45 patients with PCNSL, Ki-67 index ≥ 90% was an independent predictor of poor OS prognosis in the entire cohort as well as in the non-GCB tumor subtype (Ki-67 index = 91.1%) [223].

#### p27

P27 is a cyclin-dependent kinase inhibitor that controls the progression of the cell cycle from G1 to S phase [338]. Kunishio et al*.* employed immunohistochemistry to examine p27 expression in 22 PCNSL patients. High p27 expression was found to be highly related to shorter OS, implying that p27 might be used to predict the prognosis of PCNSL patients [338].

#### Histone methylation abnormality

Numerous genetic alterations in cancer are associated with chromatin and epigenetics, particularly histone-modified proteins. Histone modifications have a crucial role in both normal cell function and malignancy. Common modifications of histones include methylation, acetylation, ubiquitination, and phosphorylation [339]. Histone modifications have a crucial role in malignancy. Researchers immunohistochemically stained FFPE samples from 87 PCNSL patients identified by pathology. Patients with H3K4me3 hypomethylation and H3K27me2 and H3K27me3 hypermethylation were more likely to relapse. In both univariate and multivariate studies, these three variables were statistically related with a short PFS and OS. It was shown that low methylation of H3K4me3 and high methylation levels of H3K27me2 and H3K27me3 may be linked to a poor prognosis in PCNSL patients [340].

#### PD-1, PD-L1, and PD-L2

Expression levels of PD-1, PD-L1 and PD-L2 on PCNSL tumor cells can be utilized to predict patient prognosis. Takashima Y et al*.* performed RNA sequencing on samples from 31 PCNSL patients and found that changes in the expression of *PD-1* and *PD-L2* transcripts enable prognostic prediction in PCNSL. High *PD-1* (PDCD1-001: HR = 3.3, *p* = 0.012, PDCD1-002: HR = 9.3, *p* = 8.4E-05, and PDCD1-003: HR = 2.6, *p* = 0.032) and PD-L2 (PDCD1LG2: HR = 2.9, *p* = 0.018) gene expression was associated with a shorter OS [341]. Cho et al*.* analyzed the prognosis of 76 patients with PCNSL who received an HD-MTX-based chemotherapy regimen at the time of first diagnosis. The multivariate analysis revealed that high PD-1 expression (70 cells/high power field) was associated with a worse OS and a PFS [342]. Analysis of PD-L1 expression in serum and FFPE tissues of PCNSL patients revealed that the median level of serum PD-L1 was greater than that of healthy control patients; PD-L1 expression of positive tumor cells in FFPE tissues was positively correlated with serum PD-L1 level. Notably, the high serum PD-L1 group was more susceptible to recurrence than the low serum PD-L1 group [343].

The tumor microenvironment, in addition to tumor cells, influences PCNSL prognosis. Using immunohistochemistry techniques, Furuse et al*.* evaluated intratumoral and peritumoral tissues from 70 patients with PCNSL. It was discovered that a greater proportion of macrophages than tumor cells expressed PD-L1 and PD-L2. PD-L1 expression on macrophages was linked to biological factors (intratumoral macrophages: better KPS, better MSKCC score, and peritumoral macrophages: low proportion of LDH elevation) and a longer OS correlation [344]. Another study also confirmed that the increased number of PD-L1-expressing immune cells, like tumor-infiltrating lymphocytes and tumor-associated macrophages, is associated with better disease-free survival in PCNS-DLBCL [345].

#### Ku80

Ku80 is a DNA repair protein connected with radiosensitivity and plays a crucial role in multiple processes that protect against ionizing radiation. In a study reviewing 38 patients with PCNSL, Ku80 expression in tumor tissue was found to be present in most PCNSL tissues using immunohistochemistry. According to survival analysis, patients with high Ku80 expression had significantly shorter median survival times than patients with low Ku80 expression (*p* = 0.036). Intriguingly, although Ku80 was connected with radiosensitivity, it was not statistically significant when comparing the OS of patients treated with and without radiotherapy (*p* = 0.131). Consequently, Ku80 is anticipated to be a prognostic predictor for PCNSL [346]. Due to the small number of patients described in this study (n = 38), the conclusion that Ku80 cannot be used to predict radiotherapy efficacy requires further validation.

#### CD105

CD105 is a receptor for transforming growth factor (TGF)-beta1 and -beta3, and its interaction with TGF-beta receptors I and/or II modulates TGF- signaling [347–353]. Furthermore, CD105 is a proliferation-associated hypoxia-inducible protein that is overexpressed on proliferating endothelial cells engaged in tumor angiogenesis but is low or not expressed in normal tissues' vascular endothelial cells [349].

The current study investigated the link between CD105 expression and PCNSL prognosis using immunostaining for CD105. Intratumoral microvascular density (IMVD) was measured in the hotspots and interfaces at a magnification of × 200. When CD105 was utilized as an angiogenesis marker, the lower-IMVD group had a significantly greater survival rate than the higher-IMVD group. The IMVD was larger in the hotspots than in the interfaces in the group with CD105-immunostained vasculature. These findings revealed that PCNSL growth depended on angiogenesis and that IMVD, measured by an anti-CD105 monoclonal antibody, was a reliable prognostic marker in PCNSLs [354].

#### Glucose transporter protein type 1

The process of glucose metabolism is crucial in cancer development [355–361]. MTX resistance in PCNSL cells is possibly associated with altered aerobic glycolysis [362]. According to a Korean study, PCNSL patients expressed glucose transporter protein type 1 (GLUT1) in tumor tissues, and patients with > 20% GLUT1 positivity in lymphoma cells had shorter OS and more rapid disease progression [363]. GLUT-1 may affect the prognosis of PCNSL patients by having an impact on the mean value of fasting plasma glucose (FPG) levels. The percentage of GLUT1-positive cells was higher in patients with FPG ≥ 110 mg/dL (*p* = 0.015), while high mean value of FPG was a significant predictor for shorter survival (*p* = 0.036) [364]. The results of the current research suggest that the expression level of GLUT1 is associated with PCNSL prognosis.

#### PI3K/AKT/mTOR pathway-related proteins

Since the PI3K/AKT/mTOR pathway is aberrantly active in DLBCL and plays a role in the genesis and progression of DLBCL [365–369], researchers have also investigated its role in PCNSL. Zhang et al. found that the recurrence rate of PCNSL in the phospho-mTOR-positive group was 64.5%, which was substantially greater than in the negative group. The Kaplan–Meier survival analysis revealed shorter PFS in the phospho-mTOR and phospho-S6-positive groups, while PTEN loss was associated with a shorter OS. According to Cox regression analysis, phospho-mTOR expression was an independent predictor for shorter PFS. The results reveal that the PI3K/AKT/mTOR pathway is aberrantly active in PCNSL and linked with a poor prognosis, which may foreshadow the development of novel therapeutic targets and prognostic variables [365].

#### Interleukin-10

Interleukin-10 (IL-10) is a pleiotropic cytokine produced by T helper-2 cells, monocytes, macrophages, and B lymphocytes [370–376]. IL-10 not only has broad-spectrum anti-inflammatory effects but also promotes the expression of BCL-2 and protects malignant tumor cells from apoptosis [377]. Since IL-10 appears to activate STAT3, it contributes significantly to the development of PCNSL [378]. Increased IL-10 levels in CSF indicated poor KPS scores and reduced PFS or OS periods [377, 379, 380]. In a prospective study, CSF IL-10 levels were measured in 66 intracranial tumors, 26 of which were PCNSL and 40 of which were other brain tumors. The PCNSL levels were significantly higher than the other brain tumor levels. The level of IL-10 in the CSF was reduced in all patients after therapy but rose in most recurrence patients. Higher levels of IL-10 in CSF were linked to a shorter PFS [377]. The results suggest that IL-10 levels in the CSF may be a sensitive biomarker for differential diagnosis, early relapse monitoring, prognosis assessment, and evaluating the effectiveness of PCNSL. High level of IL-10 in CSF increases TAMs filtration in PCNSL, leading to shorter PFS (*p* = 0.04) [381].

Apart from the aforementioned biomolecules, some other biomolecules, such as PAX5 [320], FOXO1 [320] and Mismatch repair protein MSH2 [218], have also been found to be closely associated with the prognosis of PCNSL. Table 4 presents the outcomes of investigations utilizing biomolecules as PCNSL prognostic markers.

**Table 4:** Summary of studies investigating biomolecules as prognostic factors

**PMID**	**Factor**	**Retrospective or Prospective**	**Molecular levels**	**Method**	**Sample type**	**Number of patients/centers involved**	**Treatment**	**Median Age (range), year**	**PFS**	**OS**
23832112 [312]	*miR-21*	Prospective	RNA	qRT-PCR	serum	56/2	HD-MTX + WBRT	/	/	Test cohort: HR = 1.79, *p* = 0.03; Validation cohort: HR = 1.82, *p* = 0.01
25428379 [313]	*miR-21*	Prospective	RNA	qRT-PCR	serum	27/2	HD-MTX failed, and pemetrexed plus rituximab as second-line treatment	534 (35–63)	Low level: 9.0 months; High level: 5.7 months	/
30615673 [314]	*miR-181b, miR-30d, and miR-93*	Prospective	RNA	Gene miRNA 4.0 Chip and real-time PCR	specimens	27/4	HD-MTX	64 (31–76)	/	HR = 8.934, *p* = 0.0007
32101576 [315]	*miR-101*	Prospective	RNA	Gene miRNA 4.0 Chip and real-time PCR	specimens	40/4	HD-MTX	Training data set: 65 (44–76); Test data set: 69 (23–86)	/	HR = 0.00, *p* = 0.004
32101576 [315]	*miR-1202*	Prospective	RNA	Gene miRNA 4.0 Chip and real-time PCR	specimens	40/4	HD-MTX	Training data set: 65 (44–76); Test data set: 69 (23–86)	/	HR = 1.70, *p* = 0.001
26250566 [319]	*RNU2-1f*	Prospective	RNA	Real-time ‐PCR	CSF	9/1	HD-MTX	65 (42–87)	/	/
33591648 [47]	*MYC*	Retrospective	RNA	Real-time PCR	FFPE	35/1	HD-MTX	62 (26–84)	HR = 3.960, *p* = 0.016	HR = 6.117, *p* = 0.003
29937999 [320]	*MYC*	Retrospective	DNA	PCR and NGS based DNA measure (Illumina MiSeq system)	FFPE	27/4	/	66 (31–85)	/	HR = 0.305, *p* = 0.0012
28981733 [218]	C-MYC	Retrospective	Protein	Immunohistochemical	specimens	41/1	HD-MTX	63 (19–82)	< 15%: 32.8 m; ≧ 15%: 13.3 m; *p* = 0.301	< 15%: 73.3 m; ≧ 15%: 45.2 m; *p* = 0.046
34898570 [321]	C-MYC	Retrospective	Protein	Immunohistochemical	specimens	87/1	HD-MTX + WBRT	58 (32–81)	Negative: 124.5 m; Positive: 64.8 m; *p* = 0.008	Negative: 145.0 m; Positive: 14.4 m; *p* < 0.001
34339901 [237]	C-MYC	Retrospective	Protein	Meta-analysis	specimens	1739/ over 31	/	/	Relater with shorter median PFS: HR = 1.53, *p* = 0.780	Relater with shorter median OS: HR = 1.36, *p* = 0.009
32101329 [235]	C-MYC / BCL2	Retrospective	Protein	Immunohistochemical	FFPE	48/1	HD-MTX	60 (53–69)	The median PFS was: Without any features: not reached; With 1 feature: 0.8 years; With 2 features: 0.3 years; *p* < 0.0001	The median OS was: Without any features: not reached; With 1 feature: 2.3 years; With 2 features: 1.0 years *p* < 0.0001
34898570 [321]	BCL2	Retrospective	Protein	Immunohistochemical	specimens	87/1	HD-MTX + WBRT	58 (32–81)	Negative: 123.9 m; Positive: 92.9 m; *p* = 0.011	Negative: 146.4 m; Positive: 14.4 m; *p* < 0.001
29113178 [322]	BCL2	Retrospective	Protein	Immunohistochemical	specimens	89/2	HD-MTX	56 (11–85)	Positive *vs.* negative: HR = 0.649 *p* = 0.438	Positive *vs.* negative: HR = 0.549 *p* = 0.427
34339901 [237]	BCL2	Retrospective	Protein	Meta-analysis	specimens	1739/ over 31	/	/	Short median PFS: HR = 1.22, *p* = 0.249	Short median OS: HR = 1.05, *p* = 0.054
28981733 [218]	BCL6	Retrospective	Protein	Immunohistochemical	specimens	41/1	HD-MTX	63 (19–82)	< 30%: 13.3 m; ≧ 30%: 78 m; *p* = 0.051	< 30%: 65.1 m; ≧ 30%: 103.8 m; *p* = 0.055
34898570 [321]	BCL6	Retrospective	Protein	Immunohistochemical	specimens	87/1	HD-MTX + WBRT	58 (32–81)	Negative: 128.1 m; Positive: 91.4 m; *p* = 0.102	Negative: 135 m; Positive: 40.4 m; *p* = 0.084
33365186 [219]	BCL6	Retrospective	Protein	Immunohistochemical	specimens	86/1	/	55 (22–82)	/	Negative: 58 m; Positive: 28 m; *p* = 0.091
29113178 [322]	BCL6	Retrospective	Protein	Immunohistochemical	specimens	89/1	HD-MTX	56 (11–85)	Positive *vs.* negative: HR = 0.736, *p* = 0.571	Positive *vs.* negative: HR = 0.612 *p* = 0.468
34339901 [237]	BCL6	Retrospective	Protein	Meta-analysis	specimens	1739/ over 31	/	/	Shorter median PFS: HR = 1.04, *p* = 0.000	Short median OS: HR = 0.88, *p* = 0.055
30227305 [58]	*CD79b* mutation	Retrospective	DNA	NGS based DNA measure (Illumina MiSeq system)	FFPE	23/1	/	56 (17–78)	Related with shorter median PFS, *p* = 0.044	/
27161435 [329]	*MyD88*^*L265P*^ mutation	Retrospective	DNA	PCR	Peripheral blood	42/1	/	69 (58–82)	HR = 2.770, *p* = 0.0303	HR = 2.903, *p* = 0.0474
29258950 [330]	*MyD88*^*L265P*^ mutation	Retrospective	DNA	PCR	Frozen tissue	19/8	HD-MTX	> 65 (/)	/	Mutation: 11.4 m; Wild type: 56.2 m; *p* = 0.035
34377990 [328]	*MyD88*^*L265P*^ mutation	Retrospective	DNA	PCR	Frozen tissue	57/1	/	66 (31–78)	/	HR = 0.245, *p* = 0.004
30227305 [58]	MyD88 expression	Retrospective	Protein	Immunohistochemical	specimens	57/1	/	56 (17–78)	/	Low: 31 m; High: 8 m; *p* < 0.001
30729282 [335]	*ABCB1*^*rs1045642*^ genotype	Prospective	DNA	PCR	Peripheral blood	91/1	HD-MTX	55 (24–74)	CC genotype: 16 months; TT/CT genotype: 27 months; *p* = 0.020	CC genotype: 280 months; TT/CT genotype: 58 months; *p* = 0.292
29113178 [322]	Ki-67	Retrospective	Protein	Immunohistochemical	FFPE	89/2	HD-MTX	56 (11–85)	Ki-67 (> 90%) was associated with a shorter PFS, *p* = 0.039; Ki-67 (> 90 *vs.* ≤ 90%): HR = 0.437; *p* = 0.075	Ki-67 (> 90%) was associated with a shorter OS, *p* = 0.037; Ki-67 (> 90 *vs.* ≤ 90%): HR = 0.414; *p* = 0.162
27490760 [223]	Ki-67	Retrospective	Protein	Immunohistochemical	FFPE	45/1	/	58 (34–86)	Ki-67 ≥ 90%: HR = 4.125, *p* = 0.016	Ki-67 ≥ 90%: HR = 2.408, *p* = 0.1
18095123 [338]	p27	Retrospective	Protein	Immunohistochemical	FFPE	22/1	/	63 (47–80)	/	High p27 expression associated with poorer OS (*p* = 0.0011)
34793663 [340]	hypomethylation of H3K4me3	Retrospective	Protein	Immunohistochemical	FFPE	87/1	HD-MTX	58 (32–81)	HR = 6.31, *p* = 0.006	HR = 8.32, *p* = 0.002
34793663 [340]	hypermethylation of H3K27me2	Retrospective	Protein	Immunohistochemical	FFPE	87/1	HD-MTX	58 (32–81)	HR = 12.82, *p* < 0.001	HR = 4.05, *p* = 0.019
34793663 [340]	hypermethylation of H3K27me3	Retrospective	Protein	Immunohistochemical	FFPE	87/1	HD-MTX	58 (32–81)	HR = 10.27, *p* = 0.001	HR = 4.74, *p* = 0.011
31292525 [341]	*PD-1*	Retrospective	RNA	NGS based RNA measure (Illumina HiSeq2000/2500)	Specimens	31/3	/	67 (31–85)	/	PDCD1-001: HR = 3.3, *p* = 0.012; PDCD1-002: HR = 9.30, *p* = 8.4E-05 PDCD1-001: HR = 2.6, *p* = 0.032
29152083 [342]	PD-1	Retrospective	Protein	Immunohistochemical	FFPE	76/1	HD-MTX	57 (33–79)	HR = 2.73, *p* = 0.028	HR = 4.95, *p* = 0.007
31292525 [341]	*PD-L1*	Retrospective	RNA	NGS based RNA measure (Illumina HiSeq2000/2500)	Specimens	31/3	/	67 (31–85)	/	HR = 0.94, *p* = 0.176
28982883 [382]	PD-L1	Retrospective	Protein	Immunohistochemical	FFPE (tumor cells)	64/1	HD-MTX	64 (31–85)	/	High PD-L1expression associated with better OS (*p* = 0.0177)
32054467 [343]	sPD-L1	Retrospective	Protein	ELISA	serum	68/1	HD-MTX	55 (20–77)	High sPD-L1expression associated with better PFS (*p* = 0.008)	High sPD-L1expression associated with better OS (*p* = 0.017)
32054467 [343]	PD-L1	Retrospective	Protein	Immunohistochemical	FFPE	68/1	HD-MTX	55 (20–77)	/	*p* = 0.130
32248797 [344]	PD-L1	Retrospective	Protein	Immunohistochemical	FFPE (tumor cells)	70/6	/	68 (/)	/	*p* = 0.3523
32248797 [344]	PD-L1	Retrospective	Protein	Immunohistochemical	FFPE (intertumoral macrophages)	70/6	/	68 (/)	/	High: 60 m; Low: 24 m; *p* = 0.0328
32248797 [344]	PD-L1	Retrospective	Protein	Immunohistochemical	FFPE (peritumoral macrophages)	70/6	/	68 (/)	/	High: 60 m; Low: 14 m; *p* = 0.0061
34862664 [345]	PD-L1	Retrospective	Protein	Immunohistochemical	FFPE (immune cells)	44/1	/	55 (33–57)	*p* = 0.037	*p* = 0.006
31292525 [341]	*PD-L2*	Retrospective	RNA	NGS based RNA measure (Illumina HiSeq2000/2500)	Specimens	31/3	/	67 (31–85)	/	HR = 2.9, p = 0.018
32248797 [344]	PD-L2	Retrospective	Protein	Immunohistochemical	FFPE (tumor cells)	70/6	/	68 (/)	/	*p* = 0.3147
32248797 [344]	PD-L2	Retrospective	Protein	Immunohistochemical	FFPE (intertumoral macrophages)	70/6	/	68 (/)	/	*p* = 0.9814
23788963 [346]	Ku80	Retrospective	Protein	Immunohistochemical	FFPE	38/2	/	62 (11–82)	High: 58.6 m; Low: 79.2 m; *p* = 0.046	High: 55.3 m; Low: 80.4 m; *p* = 0.036
17102906 [354]	CD105	Retrospective	Protein	Immunohistochemical	FFPE	26/1	/	64 (48–83)	/	5-year survival rate of the lower group was significantly higher than that for the higher group (*p* < 0.01)
21281236 [363]	GLUT1	Retrospective	Protein	Immunohistochemical	specimens	51/1	HD-MTX	52 (19–77)	< 20% association with better PFS (*p* = 0.006)	< 20% association with better OS (*p* < 0.001)
35184749 [365]	phospho-mTOR	Retrospective	Protein	Immunohistochemical	specimens	43/1	HD-MTX	59 (16–78)	Negative association with better PFS (*p* = 0.002)	Negative association with better OS (*p* = 0.103)
35184749 [365]	phospho-S6	Retrospective	Protein	Immunohistochemical	specimens	43/1	HD-MTX	59 (16–78)	Negative association with better PFS (*p* = 0.009)	Negative association with better OS (*p* = 0.148)
35184749 [365]	PTEN loss	Retrospective	Protein	Immunohistochemical	specimens	43/1	HD-MTX	59 (16–78)	Negative association with better PFS (*p* = 0.218)	Normal group association with better OS (*p* = 0.072)
33240408 [380]	IL-10	Prospective	Protein	Routine biochemical	CSF	35/1	HD-MTX	56 (52–63)	CSF IL-10 levels > 1000 pg/ml related to shorter PFS	CSF IL-10 levels > 1000 pg/ml related to shorter OS
27156226 [379]	post-treatment IL-10	Prospective	Protein	Flow cytometry	CSF	79/2	HD-MTX	63 (36–88)	HR = 4.6, *p* = 0.001	/
22156547 [377]	IL-10	Prospective	Protein	ELISA	CSF	24/1	/	65 (36–83)	Elevated IL-10 level had shorter OS (HR = 3.37, *p* = 0.038)	Elevated IL-10 level had shorter OS (HR = 3.58, *p* = 0.050)
29937999 [320]	*PAX5*	Retrospective	DNA	PCR and NGS based DNA measure (Illumina MiSeq system)	FFPE	27/4	/	66 (31–85)	/	HR = 0.05, *p* = 0.0307
29937999 [320]	*FOXO1*	Retrospective	DNA	PCR and NGS based DNA measure (Illumina MiSeq system)	FFPE	27/4	/	66 (31–85)	/	HR = 0.15, *p* = 0.0278
28981733 [218]	MSH2	Retrospective	Protein	Immunohistochemical	specimens	41/1	HD-MTX	63 (19–82)	< 60%: 13.3 m; ≧ 60%: 71.6 m; *p* = 0.003	< 60%: 29.7 m; ≧ 60%: 86.9 m; *p* = 0.001

*ABCB1* ATP Binding Cassette Subfamily B Member1, *CSF* Cerebrospinal fluid, *EFS* Event-free survival, *ELISA* Enzyme-linked immunosorbent assay, *FFPE* Formalin-fixed paraffin-embedded, *GLUT* Glucose transporter protein type, *H3K27* Histone H3 lysine 27, *H3K4* Histone H3 lysine 4, *HD-MTX* High-dose methotrexate, *m* month, *MiR* microRNAs, *MYD88* Myeloid differentiation major response gene, *NA* Not mentioned, *NGS* Next-generation sequencing, *OS* Overall survival, *PCR* Polymerase chain reaction, *PD-1* Programmed cell death-1, *PD-L1* Programmed death-ligand 1, *PD-L2* Programmed death-ligand 2, *PFS* Progression-free survival, *qRT-PCR* quantitative reverse transcription-PCR, *RT-PCR* Real time-PCR

### Challenges and future perspectives

**Fig. 4:**
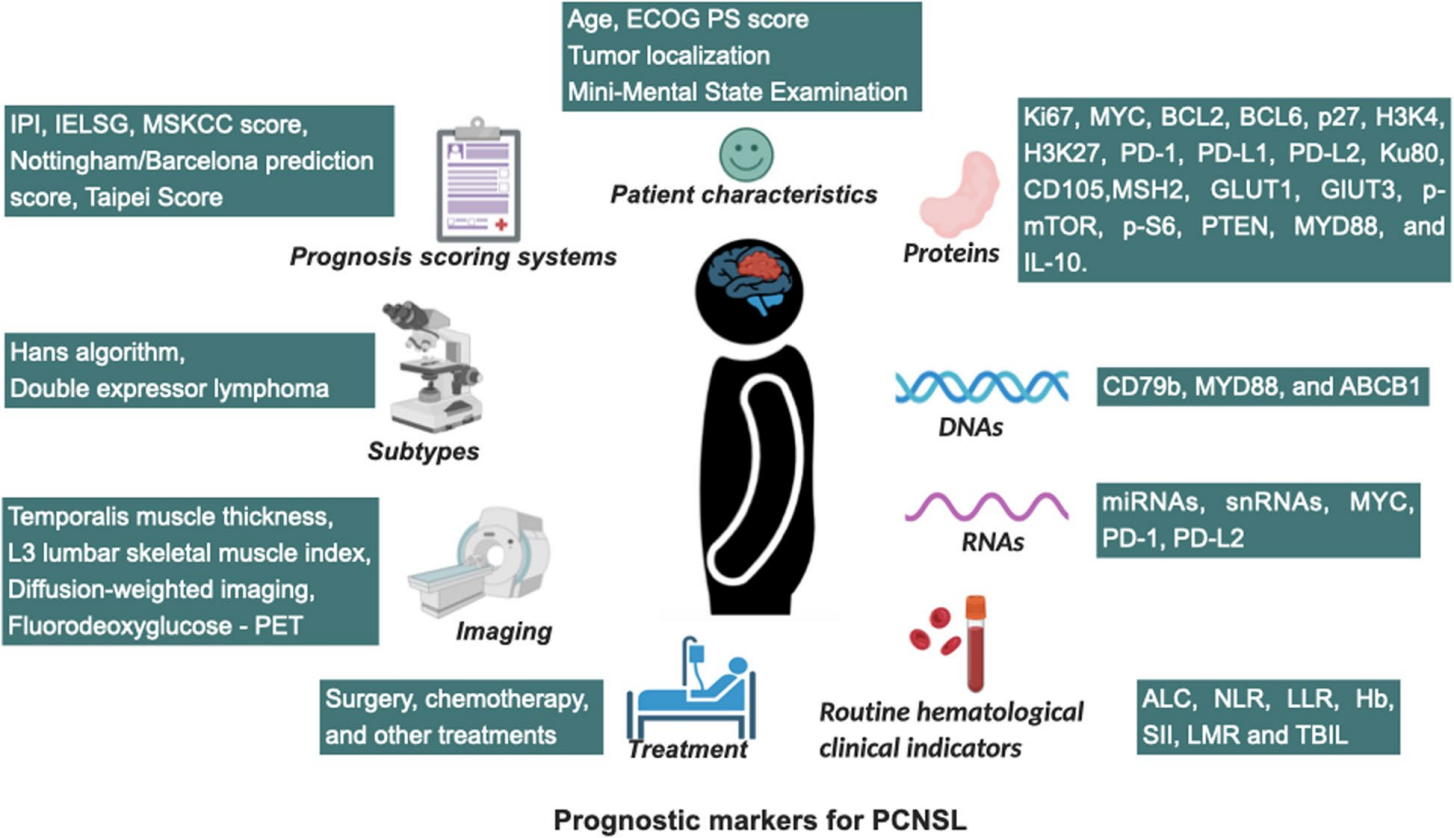
An overview of the prognostic factors currently utilized to predict PCNSL. Abbreviations: ABCB1, ATP Binding Cassette Subfamily B Member 1; ALC, Lymphocyte count; ECOG PS, The Eastern Cooperative Oncology Group Performance Status; GLUT, Glucose transporter protein type; H3K27, Histone H3 lysine 27; H3K4, Histone H3 lysine 4; Hb, Hemoglobin; IELSG, International Extranodal Lymphoma Study Group; IL-10, Interleukin-10; IPI, International Prognostic Index; LLR, Lactate dehydrogenase/lymphocyte ratio; LMR, Lymphocyte/monocyte ratio; miRNA, MicroRNA; MSKCC, Memorial Sloan-Kettering Cancer Center score; MYD88, Myeloid differentiation major response gene; NLR, Neutrophil/lymphocyte ratio; PD-1, Programmed cell death-1; PD-L1, Programmed cell death-ligand1; PD-L2, Programmed death-ligand 2; SII, Systemic immune inflammatory index; snRNA, Small nuclear RNA; TBIL, Total bilirubin

Currently, multiple prognostic markers are applied to predict the prognosis of PCNSL patients (Fig. 4). Basic PCNSL patient characteristics, imaging, treatments and subtypes help determine PCNSL prognosis. However, the sensitivity, specificity, and survival benefit of the predictors are usually unsatisfactory for routine screening.

The prognosis scoring systems are commonly used in clinical practice, and therefore receive the most attention. For IPI scoring, the classification of PCNSL ‘clinical staging’ directly impacts on the patients’ scores. Some researchers classified PSNCL as grade I-II (tumors all located on the same side of the diaphragm), while other researchers classified PCNSL as grade IV (diffuse or disseminated involvement of one or more extra-lymphatic organs). This difference in classification may affect the accuracy of IPI scoring.

Nottingham/Barcelona prediction score, which is modified form of the IPI score, and the Taipei score are used less frequently. Therefore, the accuracy of assessing the prognosis of PCNSL using the above scoring systems needs large population validation.

Currently, the internationally recognized and more widely used score for assessing PCNSL prognosis in clinical practice are the MSKCC and the IELSG score. The MSKCC prognostic model has a selection bias due to the reason that the study population is usually from the same institution [257].

IELSG score sometimes cannot be performed in PCNSL patients due to the lack of CSF protein results. Patients with PCNSL show occupying intracranial lesions with perifocal edema, and are at risk of increased intracranial pressure and potential complications. Therefore, lumbar puncture is not always performed in routine clinical practice [207].

In addition, both the MSKCC and the IELSG score are based on retrospective studies, and the treatment regimens of most patients are based on HD-MTX. With the advances in the clinical management of PCNSL, such as the widespread use of MATRix protocols, these models may not always be applicable to today's PCNSL treatment paradigm.

In recent years, more prognostic studies are performed based on laboratory hematological tests. Clinically used hematological markers, such as ALC, LLR, NLR and Hb are cost-effective, easily accessible, and to some extent, can reflect patient treatment and prognosis. However, it should also be noted that they are not as sensitive and specific for PCNSL. These indicators are susceptible to tumor comorbidities and complications, such as anemia, cachexia, chronic inflammation, and organ insufficiency.

C-MYC and BCL2 are two of the most studied proteins as they are associated with the DEL subtype classification of PCNSL. The DEL subtype can not only predict the PCNSL prognosis alone, but can also be combined with the IPI score to improve the prediction accuracy. The significance of new prognostic markers (*e.g.,* RNA, DNA and proteins) in assessing PCNSL prognosis is being investigated. The factors that currently have clear prognostic significance for PCNSL include *MYC, PD-1, MyD88*^*L265P*^ mutation, ki67, PD-1, c-MYC and IL-10 in CSF. To date, the prognostic efficacy of most factors is controversial. The role of some key factors in predicting PCNSL prognosis is unclear, such as the proto-oncogene serine/threonine (Ser/Thr) protein kinase 1 (PIM1), a known target for somatic hypermutation mechanisms in PCNSL [150, 320, 383].

Since the presented prognostic biomarkers or models for PCNSL are still unsatisfactory; new effective prognostic biomarkers and/or models are required to assist clinicians in determining the clinical progression of PCNSL and achieving more accurate therapeutic stratification.

Firstly, combining traditional tests with existing prognostic models can improve the accuracy of PCNSL prognosis. For example, the MSKCC score combined with LLR can effectively improve the accuracy of prognostic assessment in low and intermediate-risk groups [258]. Secondly, new body fluid biopsy techniques (including circulating tumor DNA, circulating tumor cells, cell-free RNA, tumor cultured platelets and exosomes) should be considered to be included in PCNSL prognostic models. The potential utility of liquid biopsy for early detection and management of cancer has emerged as a promising alternative way over traditional tissue sampling methods [384]. Thirdly, some prognostic genes, including somatic mutations, copy number variants, fusion gene alterations, may have an impact on PCNSL prognosis. Taking *MYD88* as an example, it can influence PCNSL prognosis through both aberrant expression and mutations. Future studies require subgroup analysis based on marker variants. Moreover, almost all current prognostic studies are retrospective. Prospective studies are needed to aid better stratification of PCNSL patients, and assessing the technical robustness and reproducibility of the proposed biomarkers by implementing stringent inclusion and exclusion criteria, so that patient inconsistency can be reduced. Besides, multicenter studies should be conducted through international collaborations. To improve the accuracy of study results, large-scale, forward-looking studies are needed. Finally, some novel factors have been found to express in specific PCNSL populations. For sample, N-linked oligosaccharides [385], PI3K/AKT/mTOR [362] pathway and oxidative stress [362] have been reported in relapsed or MTX-resistant PCNSL patients. PCNSL prognostic models should be developed in the future for EBV-positive, HIV-positive and rituximab populations, as well as for populations with alternative treatment methods (such as BTK inhibitors, proteasome inhibitor [386], and hematopoietic stem cell transplantation). However, due to low prevalence of PCNSL, multicenter, large-scale population and prospective studies of prognostic factors should require global collaboration.

With advances in testing technology and the development of large-scale, multicenter, prospective and international collaborative clinical studies, the technical challenges of testing PCNSL samples and the problem of biased patient data selection have been gradually overcome. New prognostic assessment models are expected to enter the clinics to assist clinicians in their decision-making.

## Conclusion and prospect

In summary, the pathogenesis of extranodal lymphoma involves a variety of mechanisms, including genetic alterations, immune dysregulation and viral infection. Viral infections are an important causative factor in extranodal lymphoma, including HP, EBV, HBV, HCV and HIV [387]. The pathogenesis of extranodal lymphoma of B-cell, T-cell and NK-cell origin varies widely. The exact pathogenesis of extranodal lymphoma is still being explored and is thought to be a complex interplay of environmental and genetic factors.

Extranodal lymphoma differs from common diseases at the site of origin or secondary lymphoma involving that site, but its clinical presentation and imaging features are often nonspecific. Therefore, a pathological biopsy is required to confirm the diagnosis. The diagnostic process involves evaluating the location, pathological type, extent, stage, immunophenotype, molecular biology, and patient-related factors of the disease. Tissue biopsy and immunohistochemistry are most important in determining the specific subtype and cell origin. Staging and risk stratification are crucial for designing an appropriate treatment plan.

The treatment approach depends on the stage and subtype of the disease. For localized disease, radiotherapy or chemotherapy alone may be effective in some cases. However, for advanced or disseminated disease, a combination of radiotherapy and chemotherapy is typically recommended. Conventional chemotherapy alone has limited success in relapsed/refractory cases. ASCT after achieving remission can benefit selected patients, while allogeneic transplantation is being explored for refractory cases.

Individualised treatment based on pathogenesis is important in extranodal lymphoma. Clinical trials have relatively focused on inhibitors targeting the PI3K/Akt/mTOR, PD-1/PD-Ls, and BCR pathways, showing promising results in relapsed/refractory extranodal lymphoma. In addition to specific pathway inhibitors, pan-pathway inhibitors are also being extensively studied. For example, MS-553, a protein kinase C (PKC) inhibitor, can act on several classical signaling pathways, such as the PI3K/Akt/mTOR pathway, the MEK/ERK pathway, and the NF-κB pathway [388]. CTLA-4 inhibitors, DNA methyltransferase inhibitors, chimeric antigen receptor T-cell therapy are also being explored in relapsed/refractory lymphomas, and there is hope for future use in extranodal lymphomas as well [389].

In conclusion, a comprehensive approach combining radiotherapy, chemotherapy, targeted therapy, immunotherapy, and transplantation offers the best chance for successful management of extranodal lymphoma. Further research is needed to better understand the underlying mechanisms and optimize treatment strategies for this complex disease.

## Data Availability

Not applicable.

## Abbreviations

ABCB1: ATP Binding Cassette Subfamily B Member 1
ADC: Apparent diffusion coefficient
AKT: Protein kinase B
ALC: Lymphocyte count
AUC: Area under the ROC curve
ASCT: Autologous stem-cell transplant
BAFF: B-cell activating factor
BCR: B-cell receptor
BTK: Bruton's tyrosine kinase
CAR-T: Chimeric antigen receptor T-cells
CDK: Cyclin-dependent kinase
CNS: Central nervous system
CSF: Cerebrospinal fluid
CR: Complete response
C-index: Harrell’s concordance index
DEL: Double expressor lymphoma
DLBCL: Diffuse large B-cell lymphoma
DSS: Disease-specific survival
DWI: Diffusion-weighted MRI
EBV: Epstein-Barr virus
ECOG PS: The Eastern Cooperative Oncology Group Performance Status
EFS: Event-free survival
FDG: Fluorodeoxyglucose
FFPE: Formalin-fixed and paraffin-embedded
FPG: Fasting plasma glucose
GCB: Germinal center B-cell
GFR: Growth factor receptors
GLUT: Glucose transporter protein type
GO: Gene Ontology
GSEA: Gene Set Enrichment Analysis
H3K27: Histone H3 lysine 27
H3K4: Histone H3 lysine 4
Hb: Hemoglobin
HD-MTX: High-dose methotrexate
HP: H. pylori
HR: Hazard ratio
IELSG: International Extranodal Lymphoma Study Group
IL-10: Interleukin-10
IL1R: Interleukin 1 receptor
IMVD: Intratumoral microvessel density
IPI: International Prognostic Index
JAK/STAT: Janus-associated kinase/signal transducer and activator of transcription
KEGG: Kyoto Encyclopedia of Genes and Genomes
KPS: Karnofsky performance score
L3-SMI: L3 lumbar-skeletal muscle index
LDH: Lactate dehydrogenase
LLR: Lactate dehydrogenase/lymphocyte ratio
LMP1: Latent membrane protein 1
LMR: Lymphocyte/monocyte ratio
miRNA: MicroRNA
MALT: Mucosa-associated lymphoid tissue
MF: Mycosis fungoides
MHC: Major histocompatibility complex
MMSE: Mini-Mental State Examination
MSKCC: Memorial Sloan-Kettering Cancer Center score
MTOR: Mammalian target of rapamycin
MYD88: Myeloid differentiation major response gene
NF-κB: Nuclear factor-kappaB
NGS: Next-generation sequencing
NLR: Neutrophil/lymphocyte ratio
NKTCL-NT: Natural killer/T-cell lymphoma (nasal type)
OS: Overall survival
PCNSL: Primary central nervous system lymphoma
PD-1: Programmed cell death-1
PD-L1: Programmed cell death-ligand1
PD-L2: Programmed death-ligand 2
PFS: Progression-free survival
PI3K: Phosphatidylinositol-3 kinase
PTCL: Peripheral T cell lymphoma
RNU2-1f: U2 small ribonucleic acid fragment
SEER: Surveillance, Epidemiology, and End Results
SII: Systemic immune inflammatory index
snRNA: Small nuclear RNA
SUV: Standardized uptake value
TBIL: Total bilirubin
TCR: T-cell receptors
TLR: Toll-like receptor
TGF: Transforming growth factor
Th: T-helper
TMT: Temporalis muscle thickness
TNFR: Tumor necrosis factor-α receptor
WBRT: Whole brain radiotherapy
